# Recent Advances in Supercritical CO_2_ Extraction of Pigments, Lipids and Bioactive Compounds from Microalgae

**DOI:** 10.3390/molecules28031410

**Published:** 2023-02-02

**Authors:** Soultana Tzima, Ioulia Georgiopoulou, Vasiliki Louli, Kostis Magoulas

**Affiliations:** Laboratory of Thermodynamics and Transport Phenomena, School of Chemical Engineering, National Technical University of Athens (NTUA), Zografou Campus, 15780 Athens, Greece

**Keywords:** microalgae, supercritical fluid extraction, lipids, pigments, bioactive compounds, green solvents, biomass pretreatment, kinetic studies

## Abstract

Supercritical CO_2_ extraction is a green method that combines economic and environmental benefits. Microalgae, on the other hand, is a biomass in abundance, capable of providing a vast variety of valuable compounds, finding applications in the food industry, cosmetics, pharmaceuticals and biofuels. An extensive study on the existing literature concerning supercritical fluid extraction (SFE) of microalgae has been carried out focusing on carotenoids, chlorophylls, lipids and fatty acids recovery, as well as the bioactivity of the extracts. Moreover, kinetic models used to describe SFE process and experimental design are included. Finally, biomass pretreatment processes applied prior to SFE are mentioned, and other extraction methods used as benchmarks are also presented.

## 1. Introduction

In the last few years, the need for naturally derived products with a low environmental footprint is steadily emerging [1]. For this purpose, not only green processes need to be applied, but also, feedstock that can be obtained with a neutral impact on the ecosystem is desired [2]. Biomass, such as microalgae, seems to have many advantages, mainly due to its ease of availability, either from controlled cultures, where no arable land is required, or from natural sources, for instance fresh water, marine environments and wastewater [2,3,4].

Microalgae are a diverse group of eukaryotic organisms or prokaryotic cyanobacteria, which can be cultivated autotrophically, heterotrophically or mixotrophically [5]. They can be reproduced rapidly, where, under the appropriate conditions an exponential production rate can be reached [3,5]. Also, thanks to the wide diversity of species and different cultivation protocols, it is possible to recover various components, namely, pigments, lipids, proteins and fatty acids [6,7,8]. Those ingredients find application in the pharmaceutical and food industry, as well as in the production of biofuels. Consequently, microalgae species are studied and recorded with ever-increasing interest [9]. 

Concurrently, green extraction methods have also gained research interest. New extraction protocols focus on minimizing the energy demands and the use of solvents. Preferably, non-toxic and non-flammable solvents derived from biomass are used [10]. Plenty of novel extraction processes can be used for this objective, such as microwave (MAE), ultrasound (UAE) and UV light assisted extraction. These techniques apply energy to the system enabling shorter extraction times and lower solvent consumption, while achieving high recovery rates [11,12,13]. 

Another solvent extraction method is supercritical fluid extraction (SFE) which is widely applied for the recovery of products from natural matrices. This is due to the properties of supercritical fluids which combine liquid- and gas-like behavior that favors the extraction of numerous compounds compared to conventional solvents in terms of quality and quantity. Those properties can be summarized into low viscosity, gas-like diffusion, liquid-like density, and near zero surface tension [14]. The most common solvent used for SFE is carbon dioxide (CO_2_), which is non-toxic, readily available, cost-effective, volatile, non-flammable, has low critical temperature and can be recycled during the process in order to avoid green-house effects [15,16]. As a result, the thermal and chemical degradation of extracts is avoided as CO_2_ is easily removed from them as a gas by a simple decompression [1]. Furthermore, the selectivity of SFE can be easily tuned by changing the extraction conditions, i.e., pressure and temperature, or by using a co-solvent [1,16,17]. However, its main drawback is the high equipment cost, mainly due to the high extraction pressure [18]. SFE of microalgae is frequently studied as a consequence of the variety of components that can be recovered [9]. Specifically, pigments and bioactive compounds derived from microalgae are used in the food industry, pharmaceuticals, animal feed and cosmetics, while fatty acids and lipids can be used for biofuel production [9,17]. The great research interest of SFE applications and microalgae is also depicted by the significant number of pertinent patents regarding them [19]. Until 2016, more than 150 patents regarding SFE of microalgae have been recorded, concerning both pigment and lipid extraction, laboratory and large scale application [19,20]. Indicatively, approximately 43% of patents cover pigments, of which 84% referred to carotenoids and 13% to chlorophylls, while 29% of the total concerned extraction of lipids from microalgae [20].

Among the most studied microalgae are *Chlorella* and *Nannochloropsis* for both pigment and lipid recovery, *Haematococcus* for astaxanthin and *Arthrospira (Spirulina)* for fatty acids [21,22,23]. 

The objective of the present study was to review the literature related to the recovery of valuable extracts from microalgae by SFE. The bibliographic review consists of 102 articles referring to the recovery of carotenoids, chlorophylls, tocopherols, lipids and fatty acids, the phenolic content and to the activity of extracts, e.g., antioxidant and antimicrobial. Also, other extraction methods, such as conventional extraction with an organic solvent (maceration, Soxhlet), ultrasound, and microwave assisted extraction, are presented for comparison purposes. The pretreatment processes prior to the extraction process are also reported, as well as the experimental design and kinetic models used to describe the course of extraction. In Table 1, an overview of the aforementioned information is presented for each microalgae species, helping the reader to easily focus on the detailed data of Table 2 and Table 3. 

## 2. Microalgal Products

**Figure 1:**
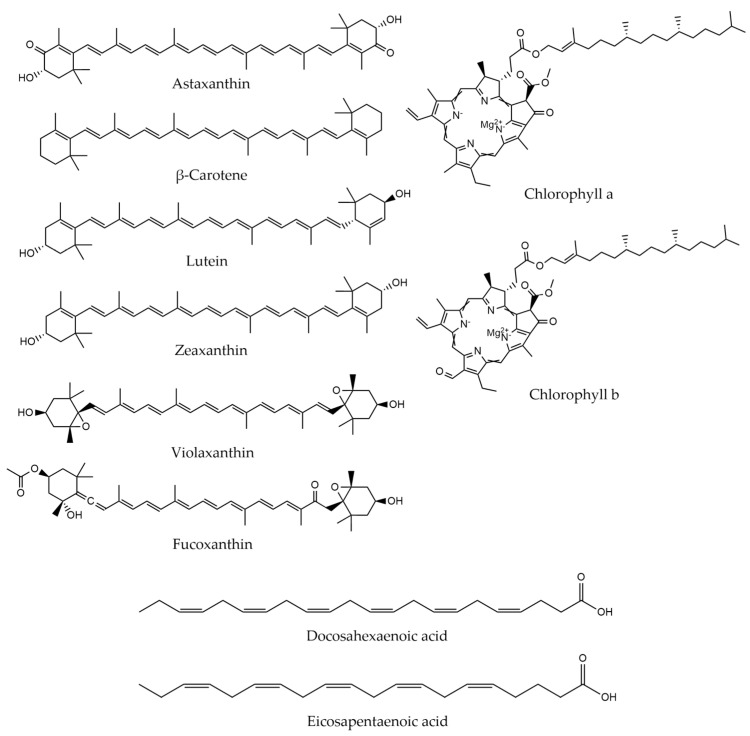
Bioactive compounds commonly found in microalgal extracts.

Microalgae is a rich source of bioactive compounds, for instance, chlorophylls, carotenoids, tocopherols and phenolics [8,126,127,128] (Figure 1). These high-added value pigments are commercially exploited to produce food supplements, pharmaceuticals and cosmetics, thanks to their antioxidant, anti-inflammatory and anti-microbial properties, among others [3,128,129]. Depending on the species and the cultivation conditions, the variety and the amount of bioactive compounds in the cells may differ [8].

Carotenoids are tetraterpenoids, soluble in lipids and responsible for the photoprotection of the microalgal cell [8,130]. They present coloring and antioxidant activities, and as a result, they are commonly used in the food industry [129,130,131]. Furthermore, carotenoids can be divided into two categories depending on the presence of oxygen in their structure [130,131]. Xanthophylls, which contain oxygen, have gained significant industrial interest for having antioxidant and conservative properties [132]. In this group, astaxanthin, lutein and fucoxanthin are included [130,131,132]. Non-oxygen containing carotenoids are called carotenes (e.g., β-carotene) [130,131,132]. Carotenoids can be categorized into primary and secondary depending on their synthesis process [130,133]. Primary carotenoids are produced during photosynthesis and are crucial for the cell’s viability, while secondary ones are produced when the cell is subdued due to stress, leading to carotenogenesis [132,133]. Factors, such as temperature, pH, salinity, light, nutrients, and the presence of oxidizing substances during cultivation may lead to an enhanced production of primary and secondary carotenoids [8]. 

In addition, chlorophylls are an extractable compound from microalgae [127,134]. Their role is to absorb solar energy, ensuring that the organism can photosynthesize [127,134]. Chlorophylls in nature may appear with plenty of isomers. The most common among microalgae is chlorophyll a, which is present in all species, while chlorophyll b is found in green algae [134,135]. Chlorophyll extracts are known for their antioxidant and antibacterial activity [127,134]. Consequently, they are widely used in pharmaceutical applications, but also, as a natural pigment due to their intense green color [127,134,135]. Their main disadvantage is that they need stabilization in order to be used as food additives, which can increase the cost and alter their beneficial properties [127].

Apart from pigments, microalgal strains also contain a significant number of fatty acids. They are carboxylic acids with compositions depending on the function they have in the cell [22]. Fatty acids can be categorized by the length of their hydrocarbon chain as short-, medium-, long- and very long-chain and by their structure as saturated (SFA), monounsaturated (MUFA) or polyunsaturated (PUFA) [22,136]. Commonly, PUFAs, such as docosahexaenoic acid (DHA), eicosapentaenoic acid (EPA) and γ-linolenic acid (GLA), are present in microalgae and find application in the food industry [6]. 

## 3. Pretreatment Methods

### 3.1. Classification of Methods

The existence of thick cell wall structures in microalgae affects the efficiency of the extraction methods. Thus, in many species, the weakening of the cell wall is necessary in order to minimize the cost of the extraction process and to enhance the recovery of target compounds [137]. There is a wide range of methods that can be used and most of them affect the microalgae cells in different ways, while aiming at extracting different compounds. Additionally, it should be mentioned that not all the pretreatments are appropriate for every process, because they alter the cell in distinct ways. Thus, the technique applied should be taken under consideration [138]. 

There are two main reasons for the necessity of a pretreatment process prior to extraction. The first one is that, in many algal species, the target compound is part of the cell wall, so by decomposing the structure, the extraction is conducted more effectively. Secondly, when extracting intracellular ingredients, the weakening of the cell wall enhances their accessibility by facilitating their transport through the cell wall [139]. The algal cell walls have tensile strength around 9.5 MPa, a fact that makes pretreatment inevitable in some cases [140].

The techniques can be briefly divided into two categories:

- Mechanical pretreatment
- Non mechanical pretreatment

The latter includes two disruption methods:

- Chemical
- Enzymatic [138]

#### 3.1.1. Mechanical

The mechanical techniques affect the cell by using shear forces, electrical pulses, waves or heat. Although they provide high recovery yields, they are not recommended for sensitive compounds due to high shear stress or temperature increases, unless a cooling mechanism is used. Their combination with other pretreatment methods may result in better recovery rates [138]. 

Bead milling is a commonly used process not only for algal biomasses, but also for grinding minerals and manufacturing paints. During this procedure, a given amount of energy is applied to the cell wall, causing the release of intracellular products. The results of pretreatment depend on bead size and type, as well as agitation speed, bead filling, chamber size and geometry, biomass concentration, and suspension flow rate [141,142].

Ultrasonication may also be performed as a biomass pretreatment technique. It can be described as a series of acoustic waves with frequencies that vary from 20 kHz up to some GHz. The waves transfer through the medium and create points with higher or lower pressure (compression or rarefaction, respectively). Those local changes, if they are intense enough, create bubbles which grow and undergo implosive collapse. This cavitation phenomenon is responsible for the ruptures caused in the cell wall surface. The energy is applied to the cell either by using an ultrasonic horn or by using an ultrasonic bath [11,143]. 

Microwaves are electromagnetic waves with frequencies between 0.3–300 GHz that generate heat depending on the polarity of the compounds. Those waves create electromagnetic fields causing the rotation of the polar compounds according to the direction of the field (dipole rotation). Respectively, ions in the medium tend to migrate with the field alternation (ionic conduction). The movement of the ions and rotation of the dipoles result in heat production by friction [11,144]. The intracellular water under microwaves evaporates, leading to an increase in pressure inside the cell and the expansion of the cell wall which causes its rupture [11].

High-pressure homogenization is a process used for sterilization and recovery of intracellular products [145]. The biomass is pumped through an orifice leading to a valve under high pressure and then expands in a lower pressure chamber. The disruption occurs because of the pressure drop which creates cavitation and shear stress on the cell wall surface [146]. The advantages of the specific method are the low heat formation, which lowers the risk of thermal degradation, and the ease of scale-up. On the other hand, for sufficient cell wall damage, a lot of circles of homogenization are required resulting in cost increases [145,147].

As in the case of ultrasonication, in hydrodynamic cavitation the cell wall ruptures because of cavitation. A Venturi valve is used in order to create a pressure drop and, therefore, cavitation bubbles which, by collapsing violently, cause damage to the cell. A major advantage of this technique is that the temperature does not increase [148,149].

The pretreatment in the case of pulsed electric field (PEF) concerns a mild disruption method because it forms pores on the cell wall for a short period of time without a significant increase in the temperature [138,150]. Specifically, when an external electric field is applied to the cell, it is believed that the lipids on the surface rearrange, enhancing the permeability of the compounds. PEF pretreatment has better results in higher cell densities, lower liquid content, and liquid systems with low cell density [151]. The conditions that affect the efficiency of the method are solvent type and dosage, temperature, and conductivity [138].

Steam explosion is a batch process where the biomass is treated under high pressure (1–3.5 MPa) and high temperature (160–260 °C). The cells are placed in a closed chamber and then the temperature and pressure are increased until the system equilibrates for 5–10 min. Afterwards, the vessel is depressurized rapidly. The sudden expansion causes the disruption of the cell wall [152]. The method is mostly used in lignocellulosic biomasses [153]. High operation temperatures might degrade thermolabile compounds, thus, lower temperatures are preferable [152].

The freeze-drying process is commonly used for drying thermolabile products in the food industry in order to maintain their quality. The procedure consists of two steps, the first is the freezing of the biomass and the second is the subjection of the sample to low pressure (approximately 1 kPa). By freeze-drying, ice crystals are formed from intracellular water, which makes the cells expand. The slower the freezing is, the larger are the crystals and the effect of the pretreatment on the biomass. A major disadvantage of the method is its high cost along with high residence time [140]. The results of this treatment are enhanced when used in combination with other methods (e.g., microwaves) [154]. 

#### 3.1.2. Chemical

A lot of materials have been used for the disruption of the cell wall. The method and the compounds used depend on the cell wall structure, its composition and the suitability with the extraction technique applied. Commonly, the substances are:

- Acids
- Solvents (organic, ionic liquids, etc.)
- Salts (e.g., osmotic shock with NaCl)
- Nanoparticles
- Surfactants [138]

#### 3.1.3. Enzymatic

Frequently, enzymatic lysis is used as a cell disruption method. Enzymes, as cellulose, break the linkage between sugars in a cellulosic chain [147]. This facilitates the extraction of the intracellular products due to their ease of accessibility through the disrupted cell wall. The method targets specific compounds depending on the enzyme used. The most used enzymes, apart from cellulose, are amylase, amyloglucosidases, lipases, and proteases [138,155]. Occasionally, a combination of enzymes in a single treatment can achieve better recovery yields [138]. Although it is an environmentally friendly procedure requiring low temperatures, the cost of the enzymes, the difficulty in scaling-up, and the slow reaction times make the method hard to apply in every case [138]. 

### 3.2. Pretreatment of Microalgae

#### 3.2.1. Arthrospira

Despite the recalcitrant cell wall of these species, pretreatments before SFE were reported only in a few studies. All the methods applied were mechanical and the majority of them involved grinding [25,26,27,39]. The rest of the pretreatments mentioned were crushing with cutting mills [28] and milling with mortar and pestle [30].

#### 3.2.2. *Chlorella*

*Chlorella* is known to have a thick cell wall, consequently, disruption methods are necessary in most cases. Frequently, milling or grinding were applied before extraction. In particular, it has been reported that disk milling increases the extraction yield from 0.076% to 0.299% in comparison with manual grinding, respectively. Adding dry ice to the manual grinding results in an extraction yield of 0.161% [58]. Also, another publication demonstrates the effect that the crushing has on the extraction yield, leading to a more than 100% increase in the yield [61]. Finally, it has been shown that by cell wall disruption with lyophilization and bead milling, a yield of 10.64% was achieved, compared to 9.25% without pretreatment [52]. Microwave pretreatment was also tested. In detail, when freeze-dried biomass was subjected to microwaves, the extraction yield increased from 3.90% to 4.86% for supercritical extraction at 28 MPa and 70 °C. More significantly though, was the effect of microwave pretreatment at lower extraction temperature, where the yield obtained was 4.73% compared to 1.81% without pretreatment [57].

#### 3.2.3. *Haematococcus*

*Haematococcus* cells, due to their rigid cell wall, when in the red non-motile stage, need to undergo pretreatment in order for carotenoids to be extracted more effectively [156]. Aravena and del Valle have studied the effect of cells homogenization with water on astaxanthin recovery [84]. Compared to powdered biomass, the homogenization leads to worse results; in particular, for extraction at 40 °C and 75 MPa, a recovery of 58% was achieved with powdered *Haematococcus*, while with homogenized cells the recovery was approximately 49% in addition to a longer extraction period. Almost the same results have been derived at 70 °C, with 61% recovery for powdered biomass and 48.5% with a water homogenized one. Nobre et al. examined the effect that the duration of the crushing has on the extracts. Under the same extraction conditions, total carotenoid recovery has been increased from 59% to 92% by doubling the crushing time [86]. Valderrama et al. achieved a yield of 0.86% at 60 °C and 30 MPa by using crushed by cutting mills biomass, while the yield reached 1.26% when crushed and manually ground with ice biomass, was extracted under the same conditions [28].

#### 3.2.4. *Nannochloropsis*

*Nannochloropsis* consists of a double layered cell wall; an external algaenan-based and an internal cellulose-based [157]. The thickness of the cell wall leads to different disruption attempts to maximize the effectiveness of the extraction method. Regarding SFE, homogenization [75,96,103] and grinding [104] have been applied to cultures. Moreover, high pressure homogenization has been tested [97]. Molino et al., have studied the outcome that accelerated solvent extraction (ASE) with n-hexane as pretreatment at 50 °C and 100 bar for 20 min [98]. Experimental design in bead-milling conditions was performed by Leone et al., focusing on the increase in extraction of lipid and total yield [106]. Microwaves seem to have a negative effect on the total recovery for the same extraction conditions since, according to Hernández et al., pretreatment for 5 minutes resulted in 8.2% yield and for 1 min in 11.9%, while the extraction yield was 12.9% when crude biomass was used [93]. Lipid yield showed different behavior, with optimum results, namely 10.8%, achieved when 1 min of microwave pretreatment was employed, while the yield was 6.9% in the case of 5 min pretreatment and 7.9% without any pretreatment. Also, water content remaining in biomass after different drying methods have been tested by Crampon et al. [102]. For freeze-drying, more humid cells resulted in higher extraction yields (same extraction conditions). Specifically, 18.4% water content resulted in 18.7% yield, while 8.5% and 4.3% water content led to 8.9% and 5.2% yield, respectively. Air dried *Nannochloropsis* with 20.4% water, yielded 22.6% and with 9.6% water content, 15.0%. Furthermore, the use of a more finely crushed biomass (<16 μm) led to a lower yield (10.3%) than that obtained with larger particles [102]. 

#### 3.2.5. *Scenedesmus*

In the case of *Scenedesmus*, all of the investigated methods were mechanical, namely microwave, ultrasonication, homogenization, bead milling and grinding. The strains were lyophilized before being subjected to cell wall disruption and/or SFE. Unfortunately, even though pretreatment is commonly applied before SFE, there are very few publications investigating its impact on the extracts. For the recovery of carotenoids and other pigments, bead-milling of the *Scenedesmus* sample before extraction resulted in significantly higher yields [111]. 

Regarding lipid extraction, microwave pretreatment positively affects the yield, in particular, it has been noted an almost double lipid yield [113]. Nevertheless, the duration of the pretreatment with microwaves seems to reduce its effect, as shown by Hernández et al. [93]. Thus, 1 minute microwave pretreatment prior to SFE resulted in a higher yield than crude biomass, while 5 minutes pretreatment led to worse results compared to non-pretreated biomass. 

Additionally, it was indicated that lyophilization as a pretreatment method does not affect FAME yields compared to fresh *Scenedesmus* samples [113]. However, it is mentioned that freeze-drying could possibly enhance the cell wall disruption in combination with other pretreatment techniques because of the increased specific area and the reduced diffusion gradient [154].

#### 3.2.6. Other Cultures

Mechanical disruption methods as a pretreatment for enhanced extraction are also applied in other species. For instance, Halim et al. have extracted *Chlorococcum,* achieving 5.8% lipid yield with dried, and then ground in ring mill biomass, compared to 7.1% with wet biomass [68]. The effect of bead-milling prior to SFE has been tested in *Pavlova* cultures resulting in 17.9% lipid yield and 15.7% FAME yield for pretreated biomass, instead of 10.4% and 5.4% for crude biomass, respectively. Furthermore, grinding has been reported by Grierson et al. for *Tetraselmis* biomass [124]. Homogenization before extraction has also been used for *Tetraselmis* by Bong and Loh [103] and for *Synechococcus* by Cardoso et al. [17] and Macías-Sánchez et al. [75]. Hernández et al. have studied the effect of microwaves as a disruption method on the extraction yield of *Tetraselmis* [93]. For crude biomass, 14.8% yield has been achieved, while for 1- and 5-min pretreatment time the extraction yield was 4.7% and 5.2%, respectively. Microwaves combined with DES in *Phaedactylum* strains have increased lipid yield from 1% without pretreatment and 5.8% when only mixed with DES, to 6.6% for 30 min at 150 °C and 7.1% for 60 min at 100 °C. Finally, Montero et al. have attempted cell wall disruption by ultrasonication, but the method did not affect the extraction efficiency [122]. 

## 4. Supercritical CO_2_ Extraction

### 4.1. Principles and Process

Supercritical Fluid Extraction (SFE) is a green process for the recovery of compounds from a solid matrix using supercritical fluids as solvents. Fluids are in supercritical state when their temperature and pressure are above critical point (T_c_, P_c_). They demonstrate properties such as low viscosity, density comparable to that of liquids, gas-like diffusion and near zero surface tension. Under these conditions, the extraction capacity of many compounds increases, therefore, supercritical fluids become a suitable solvent for a variety of applications [14]. The most commonly used solvent for SFE is supercritical CO_2_ thanks to its low critical temperature (31.1 °C) and lack of toxicity, which allows the extraction of thermolabile compounds. Moreover, Sc-CO_2_ is non-flammable, readily available, cost-effective and can be removed from the extracts by expansion to ambient conditions without any further processing, due to its gaseous state under atmospheric temperature and pressure [9,11]. Apart from that, in the supercritical region, solubility increases with the increase in density, which allows the regulation of selectivity by adjusting extraction conditions, such as temperature and pressure. For highly polar compounds, modifiers, such as alcohols, can be used in order to enhance the solubility. Furthermore, the yield and the selectivity of the process can be improved by the use of co-solvents. The above properties generate a highly selective extraction technique, resulting in extracts with high purity [11].

### 4.2. Extraction of Bioactive Compounds

#### 4.2.1. *Arthrospira*

Apart from γ-linolenic acid, which is the compound extracted in the majority of SFE applications, *Arthrospira (Spirulina)* can also provide extracts with high concentrations of carotenoids. Specifically, Canela et al. have recovered 2.27 mg/0.8 kg algae per extraction bead, at the optimal extraction conditions, namely a temperature of 30 °C, 18 MPa pressure and 11 hours extraction time [27]. Temperature, in that study, varied from 20 to 70 °C and pressure from 15 to 18 MPa. Valderrama et al. have achieved 3% phycocyanine yield and more than 97% astaxanthin recovery by extracting *A. maxima* strains at 60 °C and 30 MPa, both with and without the use of 10% *w*/*w* ethanol [28]. Similarly, experiments at 40–80 °C, 15–35 MPa and 5–15% *v*/*v* ethanol led to 48 mg/100 g_biomass_ zeaxanthin, 7.5 mg/100 g_biomass_ cryptoxanthin and 118 mg/100 g_biomass_ β-carotene yield at 35 MPa and 15% *v*/*v* ethanol [29]. Also, in another study, the maximum amount of 283 μg/g_biomass_ total carotenoids and 5.01 μg/g_biomass_ total tocopherols have been recovered from *A. platensis* at 60 °C and 450 bar with 53.22% *v*/*v* ethanol [30]. SFE on pretreated *A. platensis,* also, resulted in extract composed of approximately 290 ppm zeaxanthin, 73 ppm myxoxanthophyl fucoside, 55 ppm β-carotene and 535 ppm chlorophyll a with antioxidant activity close to 70 μg/mL (EC_50_) [34]. Additionally, Wang et al. have extracted at 48 °C, 20 MPa using ethanol as entrainer, 77.8 g β-carotene/kg_biomass_, 113.2 g vitamin a /kg_biomass_, 3.4 g α-tocopherol /kg_biomass_ and 85.1 g flavonoids /kg_biomass_ [35]. Finally, 6.84 mg/g_biomass_ chlorophyll a was recovered from *A. platensis* at 53.4 °C and 48.7 MPa with 40% aq. ethanol [37].

#### 4.2.2. *Chlorella*

*Chlorella* cultures can be used as a source of carotenoids, such as astaxanthin, canthaxanthin, lutein and β-carotene, chlorophylls and phenolic compounds. The extraction conditions, along with the use of co-solvent, can alter the extract’s composition of bioactive compounds and, thus, their antioxidant activity.

Kitada et al. have studied the effect of pressure, temperature and co-solvent on the carotenoid extraction from *C. vulgaris* [59]. Specifically, at 70 °C, 2.5 mL/min flow rate and 300 min extraction time, the lutein extracted was 0.13, 0.46, 0.40 and 0.61 mg/g_biomass_ at 20, 30, 40 and 50 MPa, respectively. The increase in temperature at a constant pressure of 30 MPa, increased the recovered lutein from 0.46 at 60 °C to 0.57 mg/g at 80 °C. The use of ethanol as co-solvent presented generally better results compared to acetone under the same conditions. Namely, 1.54 mg/g_biomass_ lutein, 0.13 mg/g_biomass_ β-carotene, 11.43 mg/g_biomass_ α-chlorophyll and 3.90 mg/g_biomass_ β-chlorophyll were recovered with ethanol and 0.94 mg/g_biomass_ lutein, 0.01 mg/g_biomass_ β-carotene, 3.30 mg/g_biomass_ α-chlorophyll and 0.59 mg/g_biomass_ β-chlorophyll were recovered with acetone. Similarly, another study indicated that the increase in pressure at 40 °C led to higher lutein recoveries. More explicitly, at 20 MPa, 1.34% lutein recovery was achieved, at 30 MPa 1.64% and at 40 MPa 1.78% [64]. Temperature increase seemed to present the opposite effect at 40 MPa, by decreasing lutein recovery to 0.67% at 80 °C [64]. The flow rate of ethanol as entrainer resulted in 1.78% lutein recovery at 0.3 mL/min, in 1.80% at 0.4 mL/min and in 1.68% at 0.5 mL/min [64]. Gouveia et al. using extraction conditions of 40 °C, 30.0 MPa and 0.0397 kg/h Sc-CO_2_, have reported maximum total carotenoid recovery of 69.1% for completely crushed *C. vulgaris* cells without the use of co-solvent, while when mixed with oil and with double the flow rate the recovery obtained was 16.6% [58]. Fairly crushed and slightly crushed cells without the use of entrainers led to a recovery of 37.3% and 17.4%, respectively. Different co-solvents showed little impact on the carotenoid recovery since 19.7% was achieved with oil and 20.2% with ethanol. Safi et al. accomplished better results in overall extract characterization for bead milled *C. vulgaris* biomass by increasing pressure from 35 MPa to 60 MPa [52]. In terms of total mass recovered, at 60 MPa pressure 10.64% yield was achieved, in contrast to 9.7% at 35 MPa. Total carotenoids and total chlorophylls reached 60 MPa 1.72 mg/g_dry biomass_ and 1.61 mg/g_dry biomass_, respectively.

Mendes et al. have investigated the effect of three operational conditions (temperature, pressure and pretreatment) on the carotenoid recovery [24]. The optimum carotenoid recovery for crude *C. vulgaris*, almost 500 mg/kg_dry algae_, was achieved at maximum temperature and pressure, i.e., 55 °C and 35 MPa. From the three degrees of crushing, whole, slightly, and well crushed, the second presented analogous results with the third, approximately 40% total carotenoids yield, but with larger requirements of Sc-CO_2_. In a similar study, under the same extraction conditions, best results were derived for the most intense extraction conditions for both crude and pretreated biomass, i.e., 171.1 mg carotenoids per 100 g oil and 0.05% *w*/*w* carotenoid yield [61,62]. Hu et al. have carried out an orthogonal experimental design that consisted of 16 experiments, where each factor consisted of four levels, in order to examine the effect of five factors (temperature, pressure, duration, Sc-CO_2_ flow rate and co-solvent quantity) on extraction yield and antioxidant capacity [46]. Yield reached its maximum value, 7.78%, at 32 °C, 40 MPa, 20 kg/h Sc-CO_2_ flow rate, 180 min and 1 mL ethanol per gram of *C. pyrenoidosa*. The inhibition at those conditions was 42.03%, while the optimum was 54.16% with 3.50% yield at 40 °C, 35 MPa, 20 kg/h Sc-CO_2_ flow rate, 150 min and 1.5 mL/g ethanol. Consequently, the most effective parameters were pressure for yield and modifier for antioxidant activity. Georgiopoulou et al. studied the SFE of *C. vulgaris* and specifically the effect of temperature, pressure and solvent flow rate on total extraction yield, antioxidant activity, total phenolic content and target carotenoid compounds, by applying experimental design [66]. The experiment under the optimum conditions (60 °C, 250 bar and 40 g Sc-CO_2_/min) resulted in 3.37% yield, 44.35 mg_extr_/mg_DPPH_ antioxidant activity using an IC_50_ assay, total phenolic content equal to 18.29 mg gallic acid/g_extract_, 35.55 mg/g_extract_ total chlorophyll content, 21.14 and 10.00 mg/g_extract_ total and selected carotenoid content, respectively. Furthermore, the addition of 10% *w*/*w* ethanol as entrainer enhanced antioxidant activity and yield. Wang et al. investigated the properties of the extract obtained by the SFE of *Chlorella* at 50 °C, 31 MPa, 6 Nl/min and the use of 50% aqueous ethanol [65]. The total polyphenol content of the extract was 13.40 mg_GAE_/g_extract_, while the total flavonoid content was 3.18 mg_QE_/g_extract_. The inhibition value in the DPPH assay was 47.24% compared to gallic acid’s 100% inhibition. In other research, in which experimental design was employed, the recovery of lutein from superfine pulverized *C. pyrenoidosa* with the use of ethanol as entrainer, reached its maximum value, 87.0% extraction yield. The conditions of that experiment were 50 °C, 25 MPa, 240 min duration and 50% *w*/*v* ethanol [47].

#### 4.2.3. *Haematococcus*

*Haematococcus pluvialis* has gained significant research interest due its high content of natural astaxanthin [158]. Yothipitak et al. have estimated that the recovery of astaxanthin could reach 22.66 mg/g_biomass_ by SFE at high pressure and temperature (64 MPa and 90 °C) [80]. SFE, with or without the use of co-solvent, appears to be an adequate technique for astaxanthin extraction, reaching, in certain cases, more than 80% recovery. Extraction of lyophilized *H. pluvialis* at 45 °C, 48.3 MPa and 2.7 mL/min Sc-CO_2_ flow rate, led to almost 85% astaxanthin recovery [85]. Likewise, 83% recovery, equal to 22.84 mg/g_biomass_, was achieved at slightly higher pressure and flow rate (50 MPa and 3 mL/min) and 80 °C [81]. Moreover, ethanol as co-solvent has been widely investigated. Bustamante et al. recovered 84% of biomass astaxanthin at 40 °C and 55 MPa with the addition of 4.5 *v*/*v* ethanol [82] and, correspondingly, Pan et al. recovered 73.9% by using 9.23 mL/g_biomass_ of aqueous ethanol under moderate conditions [83]. Similar studies of SFE at 70 °C and 40 MPa with 5% *v*/*v* ethanol led to 80.6% astaxanthin recovery [87], while at 65 °C, 43.5 MPa with 2.3 mL/g ethanol and at 55 °C, 20 MPa with 13% *w*/*w* ethanol, the recovery obtained was 87.4% and 82.3%, respectively [89,90]. SFE of powdered biomass resulted in 61% astaxanthin recovery at 70 °C and 55 MPa [84], while SFE of lyophilized and crushed *H. pluvialis* with 9.4% *w*/*w* ethanol as co-solvent led to a recovery of 92% of total carotenoids, 76% of β-carotene and 90% of astaxanthin [86]. Dried *H. pluvialis* extraction with 10% *v*/*v* olive oil as co-solvent under optimum conditions (70 °C, 40 MPa) resulted in 51% recovery of available astaxanthin [88]. Finally, extraction of red phase *Haematococcus* at 65 °C and 55 MPa resulted in high astaxanthin and lutein recoveries, 92–98.6% and 52.3–93%, respectively [91,92]

#### 4.2.4. *Nannochloropsis*

Supercritical fluid extraction of *N. gaditana* at 60 °C, 40 MPa and 4.5 mmol/min flowrate led to the recovery of 0.343 μg/mg_biomass_ total carotenoids and 2.238 μg/mg_biomass_ chlorophyll a [96] while at 50 MPa, 2.893 μg/mg_biomass_ total carotenoids, 0.369 μg/mg_biomass_ chlorophyll a and almost 0.33% total carotenoid yield were obtained [75,76]. Sánchez-Camargo et al. extracted from the same species 0.18 mg/g_biomass_ (8.3% recovery) violaxanthin at 55 °C and 40 MPa [97]. Zeaxanthin extraction from *N. oculata* was, also, carried out leading to 63.2% recovery and 13.7 mg/g_exract_ [101]. Lastly, SFE on *Nannochloropsis* sp. biomass at 40 °C and 30 MPa, with the addition of 20% *w*/*w* ethanol resulted in an extract composed of 13.71% astaxanthin, 22.35% lutein, 13.20% violaxantin and neoxanthin, 34.3% vaucheriaxanthin, 4.71% canthaxanthin, 5.08% β-carotene and 3.37% chlorophyll a [105].

#### 4.2.5. *Scenedesmus*

*Scenedesmus* cells contain both carotenoids and chlorophylls that can be recovered by SFE with or without the use of co-solvent [159]. A lutein recovery has been reported for *S. almeriansis* of 0.0466 mg/g_biomass_ at 60 °C, 400 bar and extraction duration of 300 min [111]. Also, for the same species, another study reports a recovery of 2.97 mg/ g_biomass_ of lutein for a shorter extraction time, but increased temperature and pressure, i.e., 65 °C and 550 bar [112]. The addition of a polar co-solvent in the SFE could affect the extraction of the target compounds by increasing the solvent’s polarity, and therefore, their solubility in the medium [160]. Indeed, the lutein yield seemed to have been augmented from 0.206 mg/g_biomass_ to 2.210 mg/g_biomass_ by adding 30% *v*/*v* ethanol maintaining the same temperature, pressure and time [118]. Similarly, the yield increased from 0.2105 mg/g_biomass_ lutein to 0.4361 mg/g_biomass_ with the addition of 10% *v*/*v* ethanol [119]. Remarkably, the extraction conditions which lead to the maximization of the lutein yield does not always match with the most intense ones. The same phenomenon is observed for β-carotene and lutein extraction, which both reach their maximum recovery (1.5 mg/g_biomass_ and 0.047 mg/g_biomass,_ respectively) at 60 °C, 400 bar and 300 minutes total extraction [111]. In this case, co-solvent contribution seems to be not so intense, since the use of 10% *v*/*v* ethanol led to the increase in the extracted β-carotene from 0.0547 mg to 0.0599 mg per dry biomass [119]. As a result, the best total carotenoid recovery does not occur under very intense extraction conditions. For example, SFEat 40 °C, 400 bar, and 2 h duration resulted in a recovery equal to 48.39 mg/g_extract_ and 0.303 mg/g_biomass_ at 250 bar, the same temperature and double duration [114,131]. Additionally, more carotenoids were detected, such as astaxanthin, neoxanthin, violaxanthin and zeaxanthin, and the recovery of all of them, except for violaxanthin appeared to increase with the use of co-solvent [119].

In terms of chlorophylls, they seem to have similar behavior to carotenoids. At 50 °C, 250 bar, and extraction time equal to 120 min, 15.68 mg/g_extract_ of chlorophylls were recovered [114]. Chlorophyll a is extracted in larger quantities in contrast to chlorophyll c. For example, Guedes et al. extracted 0.848 mg/g_biomass_ of chlorophyll a while chlorophyll b and c quantities obtained were 0.356 mg/g_biomass_ and 0.018 mg/g_biomass_, respectively [131].

The extraction yields reported in the various studies show significant diversity, possibly due to different species, different cultivation and different SFE conditions. The species *obliquus* presents the lowest yields among them all. The highest cited is 8.3% at 20 °C, 120 bar and 540 min total extraction time [2]. Also, SFE at 40 °C, 400 bar for 120 min resulted in 1.15% yield as reported by Gilbert-López et al. [114], while Choi et al. obtained a yield of 4.20% under almost the same conditions [115]. By the addition of 15% *v*/*v* ethanol as co-solvent, the latter yield was increased to 14.51% [115]. However, other research presented a 0.247% yield with 5% *v*/*v* ethanol at 65 °C, 300 bar and for 90 min, which deviates significantly from the results of the other researchers [44].

The SFE of the species *almeriensis* at 60 °C, 400 bar and 120 min total extraction time, led to 1.50% yield [112]. Similarly, SFE at 45 °C, 300 bar and 90 min with the addition of 5% *v*/*v* ethanol resulted in 19.4% yield [93]. The extraction of species of *obtusiusculus* at 20 °C, 120 bar and 540 min resulted in a yield of 6.4% [2]. Ultimately, SFE of unspecified *Scenedesmus* species led to yields up to 6.81% [120].

#### 4.2.6. Other Cultures

In addition to the species mentioned above, *Dunaliella salina* cultures are also a major carotenoid and chlorophyll source. Specifically, extraction carried out at 40 MPa and 60 °C recovered 12.17 μg/mg_biomass_ carotenoids and 0.227 μg/mg_biomass_ chlorophylls [74]. By using 5% mol ethanol as co-solvent, under the same conditions, the yield altered to 9.629 μg/mg_biomass_ carotenoids and 0.700 μg/mg_biomass_ chlorophylls [76]. Similarly, Pour Hosseini et al., at slightly lower temperature and without co-solvent, obtained 115.44 μg/g_biomass_ total carotenoids and 32.68 μg/g_biomass_ chlorophylls [77]. Under milder conditions, namely 45 °C and 20 MPa with 5% *w*/*w* ethanol, Molino et al. recovered 25.5% of β-carotene from *D. salina* [78]. Total carotenoid content was also determined at 27.5 °C, 44.2 MPa and 45 °C, 20 MPa and found to be equal to 7.2 mg/100 g_extract_ and 25 g/kg_biomass_, respectively [72,79].

SFE of *Chlrococcum littorale* recovered 89% of extractable carotenoids and 48% of chlorophylls [69], while SFE of *Isochrysis galbana* at 50 °C and 30 MPa led to the recovery of 16.2 mg/g_biomass_ carotenoids and 4.5 mg/g chlorophylls [94]. Chatterjee et al. determined that the total carotenoid content of *P. valderianum* was equal to 13.43 μg β-carotene equivalent/g_biomass_ at 50 °C and 50 MPa [110]. Fujii extracted from *Monoraphidium* sp. 2.46 mg/g_biomass_ astaxanthin, which is equal to 101% recovery, by using 20 mL ethanol as entrainer at 60 °C and 20 MPa [95].

Lastly, carotenoids such as β-carotene, β-cryptoxanthin and zeaxanthin were recovered from *Synechococcus* sp. Explicitly, maximum recovery 71.6%, 90.3% and 36.4%, of β-carotene, β-cryptoxanthin and zeaxanthin, respectively, was achieved [122]. Additionally, the SFE at 40 °C,40 MPa and 5% mol ethanol performed by Cardoso et al., resulted in 20.35 mg/g_extract_ β-carotene and 25.96 mg/g_extract_ zeaxanthin [17]. The addition of ethanol as co-solvent appears to have a positive effect on the pigment extraction. Macías-Sánchez et al., by using 5% mol ethanol under the same extraction conditions, achieved an increase from 1.51 to 1.86 μg/g_biomass_ in carotenoid recovery and from 0.078 to 0.286 μg/g_biomass_ in chlorophyll recovery [76,123].

### 4.3. Extraction of Lipids and Fatty Acids

#### 4.3.1. *Arthrospira*

The most common fatty acid extracted through SFE from *Arthrospira* cultures is GLA and, in general, an alcohol as co-solvent is used. GLA yield equal to 0.44% was achieved by conducting SFE of *A. maxima* at 60 °C, 35 MPa, 2 g/min solvent flow rate and 10% *v*/*v* ethanol [24,25,26]. Sajilata et al. recovered 102% GLA from *A. platensis* at 40 °C, 40 MPa and 0.7 L/min Sc-CO_2_ flow rate [32], while other research on the same species, presented 24.7% recovery at 40 °C and 30 MPa with 50% *v*/*v* ethanol [31]. Total fatty acid content was, also, determined. Andrich et al. by performing SFE of *A. platensis* at 55 °C and 70 MPa obtained a total FA content equal to approximately 40% [39]. At lower pressure, slightly increased temperature and with 53.22% *v*/*v* ethanol as co-solvent, Esquivel-Hernandez et al. recovered from the latter species, 34.76 mg/g_biomass_ fatty acid [30]. Qiuhui et al. determined the FA composition of *A. platensis* extract derived from extraction at 40 °C, 35 MPa and 24 kg/h solvent flow rate [33]. Specifically, the extract consisted of 16.91% oleic acid, 36.51% linolic acid, 16% α-linolenic acid and 19.68% γ-linolenic acid. Similarly, SFE with ethanol under optimum conditions, 48 °C and 20 MPa, led to the following extract composition: 35.32% palmitic acid, 21.66% α-linolenic acid and 20.58% linoleic acid [35]. Finally, Mendiola et al. examined the effect of temperature, pressure and the use of co-solvent on palmitic and oleic acid recovery from *A. platensis* [36].

#### 4.3.2. *Chlorella*

Solana et al. studied the composition of the extracts derived from SFE of *C. protothecoides* at 60 °C, 30 MPa and 5% ethanol, which consisted of 25.68% saturated fatty acids, 13.12% monounsaturated fatty acids, 61.77% polyunsaturated fatty acids, 15.13% Ω-3 and 23.53% Ω-6 [44]. Extraction of *C. vulgaris* at 40 °C and 37 MPa, with a mixture of hexane and ethanol as co-solvents, led to extracts composed of 30.05% palmitic acid, 30.22% stearic acid, 3.24% lauric acid, 4.82% myristic acid, 3.01% arachidic acid, 2.54% palmitoleic acid, 3.38% oleic acid, 1.63% linoleic acid, 1.71% docosahexaenoic acid and 2.98% eicosapentanoic acid [67]. Alhattab et al., by performing SFE of *C. saccharophila* at 73 °C and 24.1 MPa recovered extracts composed of 20.4% total FAME [48]. Microwave pretreated *C. vulgaris*, submitted to SFE at 70 °C and 28 MPa, led to 26.589 mg palmitic acid/ 100 mg_oil_, 27.296 mg oleic acid /100 mg_oil_, 10.403 mg linoleic acid /100 mg_oil_ and 16.163 mg α-linoleic acid /100 mg_oil_ [57].

Lipid recovery from *Chlorella* by applying SFE was mainly conducted with the use of co-solvent. In detail, SFE of *Chlorella* sp. with 5% ethanol at 60 °C and 30 MPa led to 79.53% lipid yield [54]. Also, at lower pressure while using 0.4 mL/min hexane, lipid yield was determined as 63.78% [53]. Moradi-kheibari et al. recovered from *C. vulgaris* 6.68% lipids at 45 °C, 35 MPa and 10% *v*/*w* ethanol [60]. For the same species, with 10% *v/v* ethanol, 97% of neutral lipids, approximately 25% of glycolipids and 35% phospholipids were recovered at 50 °C and 25 MPa [63]. Finally, Mendes et al. extracted 54.26 mg/g_biomass_ lipids from *C. vulgaris* at 55 °C and 35 MPa [62].

#### 4.3.3. *Nannochloropsis*

The SFE of fatty acids from *N. gaditana’s* were also studied. Molino et al. at 65 °C and 25 MPa recovered approximately 7.5 mg/g_biomass_ SFAs, 8 mg/g_biomass_ MUFAs, 10.5 mg/g_biomass_ PUFAs, 11.50 mg/g_biomass_ EPAs, while lipid yield was 34.15 mg/g_biomass_ [98]. SFE of *N. oculata* at 40 °C and 20.7 MPa resulted in extracts composed of 35% total SFAs, 45.31% MUFAs and 19.69% PUFAs [103]. FAME yield from *N. granulata* reached 18.23 mg/g_biomass_ at 70 °C and 35 MPa [99], while in another study for the same species and conditions, crude lipid yield reached 256.3 g/kg_biomass_ [100]. Crampon et al. at 60 °C and 40 MPa obtained an extract from *N. oculata* composed of 93.82% triglycerides and 1.80% sterols [38,102]. Finally, fatty acid composition of *Nannochloropsis* sp. extracts obtained at 40 °C and 30 MPa was found to be as follows: 25.3% SFAs, 20.1% monoenoic acid, 54.6% PUFAs [104].

#### 4.3.4. *Scenedesmus*

The EFA with the highest concentration in the lipid extracts of *Scenedesmus* by SFE was found to be α-linolenic acid (ALA). Specifically, for the species *obliquus,* when extracted at 45 °C and 150 bar for 30 minutes, the percentage of ALA in the extracted lipids reached 21.47% [44], while in other research it was found to be equal to 28.44% by conducting extraction at 20 °C and 120 bar for 540 min total extraction time [2]. The concentration of LA in the aforementioned cases was 10.33% and 10.21%, respectively. It should be noted that the optimum extraction conditions, regarding the highest concentration of ALA and LA in the extracts, coincide. Contrariwise, an almost four times higher concentration of LA compared to ALA in *S. obliquus* extracts obtained by SFE at 40 °C and 379 bar is reported [115]. Moreover, for the species *obstusiusculus*, less ALA and LA were recovered in comparison with *obliquus* under the same conditions [2]. *S. almeriensis* extracts, in contrary to other species, contain 2.9% LA while no ALA was detected. However, these extracts contained more EPA (7.9%) compared to those of *obliquus* and *obstusiusculus species* which had less than 0.59% [93].

#### 4.3.5. Other Cultures

SFE of *B. braunii* at 50 °C and 25 MPa resulted in an approximately 18% yield [41]. Halim et al. have extracted from *Chlorococcum* sp. a 1.4% FAME yield [68]. Lyophilized *C. cohnii*, when extracted with SFE, led to extracts composed of 72% DHA [71]. Molino et al. recovered 8.47 mg/g_biomass_ FAME (97.07% recovery) from *D. salina* at 75 °C and 55 MPa [78]. Additionally, lipid yield of SFE of *Ochromonas danica* reached 234.2 mg/g_biomass_ at 40 °C and 17.2 MPa [107].

### 4.4. Kinetic Models

The mathematical modeling of SFE in solid matrixes provides valuable information about the course of extraction. Using as independent variables, the operational conditions, such models describe the progress of the extraction over time, making the optimization and the simulation of the process possible [161,162]. The solid particles are usually depicted as spheres or cylinders and the mass transfer phenomena occurring in the biomass can be described by linear driving force models, shrinking core models, broken plus intact cell models and the combination of the latter [162]. Some hypotheses can be made in order to simplify the kinetic models, such as immobilized cells with constant density and porosity and isothermal and isobaric conditions in the extractor [162].

#### 4.4.1. Broken Plus Intact Cell Model

This model based on Lack’s plug flow model was proposed by Sovová and co-workers [161,163], and assumes that cell walls function as an additional resistance to the extraction of the solute. Grinding of the biomass results in disrupted and intact cells where the solute transfers to the supercritical phase through convection and molecular diffusion, respectively [162]. The extract primarily gets exhausted from the broken cells and gradually from the intact, resulting in three mass transfer periods. Initially, the extraction rate increases constantly and then falls progressively, ending up in a diffusion controlled period [164]. Sovová’s kinetic model was applied successfully in the SFE of various microalgal biomasses. Specifically, Mouahid et al. employed it for the SFE of *Arthrospira platensis*, *Chlorella vulgaris*, *Cylindrotheca closterium* and *Nannochloropsis oculata* [38] and Hernández et al. for *Isochrysis* sp., *Nannochloropsis gaditana*, *Tetraselmis* sp. and *Scenedesmus almeriansis* [93]. Solana et al. have studied the extraction kinetics of *Chlorella protothecoides*, *Nannochloropsis salina* and *Scenedesmus obliquus* [44]. Other studies involve *Chlorella vulgaris* [55,66], *Haematococcus pluvialis* [82] and *Nannochloropsis gaditana* [97].

#### 4.4.2. Other Models

Apart from models such as the linear driving force model (LDF) and shrinking core model, desorption, solubility based on Fick’s diffusion law models are often employed for the description of the SFE process on microalga. Examined species are *A. maxima* and *A. platensis* [25,27], *C. protothecoides* [43], *Chlorococcum* sp., *Synechococcus* sp., *D. salina*, *N. gadiatana* [75] and *Nannochloropsis* sp. [104].

## 5. Other Extraction Methods

### 5.1. Maceration

Maceration, is a commonly used method for microalgae extraction. Specifically, for *A. maxima,* maceration was conducted by using as solvent hexane, ethanol or acetone under ambient conditions in order to determine its lipid and GLA content [24,25,26]. Similarly, for *A.pacifica*, methanol with acetyl chloride as solvent was used for GLA recovery [32] and hexane for lipid yield [39]. Gouveia et al. used soy bean oil and acetone extraction for total lipid determination at 25 °C and 100 °C on *C. vulgaris* [58]. Also, the latter for the same species was determined with hexane and acetone maceration by Mendes et al. [61]. Lipid content of *Chlorococcum* sp. was specified by hexane and isopropanol/hexane extraction [68] while for *P. tricornutum* DMC was employed as solvent [109].

Hydrocarbon content of *B. braunii* was determined by using hexane [24,40]. Morcelli et al. by using ethyl acetate and methanol measured the concentration of violaxanthin, lutein and total carotenoids for *C. sorokiana* [49]. Total carotenoid content was determined by employing acetone for *C. vulgaris* [40], *N. gaditana* [97], *Nannochloropsis* sp. [105] and *S. obliquus* [116]. The latter study also estimated the extract’s composition regarding chlorophyll α, b and c. Relatedly, maceration with acetone led to astaxanthin extraction from *H. pluvialis* [84,85]. Among others, acetone was also utilized to recover lutein from *Scenedesmus* sp. [118] and *S. almeriansis* [111], as well as for the determination of total extractable compounds for *S. obliquus* [114], and for β-carotene extraction from *S. almeriansis* [111]. Other solvents, such as alcohols, were additionally used for pigment extraction. Methanol maceration was employed for total carotenoid and chlorophyll content determination in the case of *D. salina* [77]. Similarly, ethanol extractions were performed on *I. galbana* for the determination of total extractable compounds [94], on *Monoraphidium* sp. for astaxanthin and total chlorophyll recovery [95] and on *C. vulgaris* for astaxanthin, lutein, β-carotene and total chlorophyll content determination [66]. Lutein recovery from *Scenedesmus* sp. was achieved by using various solvents, such as methanol, ethanol, propanol and butanol [118]. Finally, ethyl acetate maceration was used for total carotenoid extraction from *Nannochloropsis* sp. [105] and tetrahydrofuran with methanol for zeaxanthin, β-carotene and β-cryptoxanthin recovery [29].

### 5.2. Soxhlet

The Soxhlet technique is commonly used as a reference method for the determination of total extractable content of the solid matrix. Its application to microalgae can lead to the extraction of lipids, chlorophylls and bioactive compounds. By using this method with hexane, total lipid extraction was achieved for *C. protothecoides* [43], *C. vulgaris* [55,56], *Chlorococcum* sp. [68], *N. granulata* [99], *N. oculata* [101], *Nannochloropsis* sp. [104,105], *Pavlova* sp. [108] and *Scenedesmus* sp. [120]. Additionally, FAME recovery was performed for *N. granulata* [99] and *Pavlova* sp. [108], as well as, SFA and PUFA extraction from *Nannochloropsis* sp. s [104]. The mixture of methanol/chloroform is also widely used for lipid content determination of biomass. Soxhlet extraction using methanol/chloroform was performed in the case of *C. vulgaris* to recover neutral lipids, phospholipids and glycolipids [63]. Also, free fatty acid conversion and lipid yield were determined for *Isochrysis* sp., *N. gaditana, S. almeriansis, Tetraselmis* sp. [93] and *S. obliquus* [44]. Using the latter mixture of solvents, PUFAs, MUFAs and SFAs have been recovered from *S. obliquus* [44]. Ethanol extractions were carried out in order to determine lipid yield for *Nannochloropsis* sp. [105], total carotenoid content for *N. oculata*, as well as lutein and total chlorophyll content for *C. vulgaris* [59]. Finally, astaxanthin extraction from *H. pluvialis* was examined using dichloromethane [83,87,88] and acetone [81].

### 5.3. Bligh and Dyer and Folch

Bligh and Dyer and Folch protocols are conventional extraction techniques commonly used for total lipid recovery from solid biomasses. While, originally, they were applied on fish tissues, these methods are a benchmark of lipid content determination of biological samples [165,166,167]. The mixture of chloroform, methanol and water in different proportions is usually used as solvent [167]. Modifications of the protocols, such as ultrasonication assistance, can also be performed on microalgae [32,168]. For total lipid content, the Bligh and Dyer method was carried out for *A. maxima* [24,25,26], *C. vulgaris* [61], *Chlorella* sp. [52], *C. cohnii* [71], *Nannochloropsis* sp. [105,106], *S. almeriansis* [112], *S. dimorphus* [113], *Scenedesmus* sp. [120], *Phaeodactylum tricornutum* [109] and *Tetraselmis* sp. [125]. Using hexane as solvent, lipids were also extracted from *S. obliquus* and *S. obtusiusculus* [2] and, assisted by sonication, from *Scenedesmus* sp. [120].

Fatty acids were also recovered by using the Bligh and Dyer protocol. Indicatively, total FA content was determined for *B. braunii* [41] and free FA conversion for *N. oculata* [102]. For the latter, triglycerides and sterols were extracted similarly. Additionally, total FA, polyunsaturated FA and EPA content of *Phaeodactylum tricornutum* were determined [109]. γ-Linolenic acid was extracted from *A. maxima* [25,26] and from *A. platensis,* assisted by ultrasonication [32].

### 5.4. Ultrasound Assisted Extraction

The present method is suitable for the recovery of heat-sensitive substances due to low temperatures, even ambient ones, during the extraction. Also, it has a shorter duration than conventional extraction methods and generally presents a higher yield. The process is fairly simple and the equipment required is readily available and relatively inexpensive [11]. In literature, many solvents have been used for the UAE of bioactive compounds and lipids, most of them being alcohols. Namely, methanol was used to extract carotenoids and chlorophylls from *D. salina, N. gadiatana* and *Synechococcus* sp. [74,76,96,123], while mixed with ethyl acetate, it recovered FAME and lipids from *Pavlova* sp. [108] and commercial DHA algae [70], respectively. Aqueous ethanol was employed for quercetin extraction from *C. vulgaris* [65]. Carotenoids and fatty acids were extracted using DMF. Specifically, total chlorophyll and carotenoid contents of *D. salina, N. gaditana* and *Synechococcus* sp. were determined [74,76,122], as well as, myxoxanthophyl, β-carotene, β-cryptoxanthin, zeaxanthin, oleic, linoleic, palmitic and palmitoleic acid content of the latter species [17,122].

### 5.5. Microwave Assisted Extraction

Microwave assisted extraction (MAE) is a non-conventional method which uses electromagnetic waves, with frequencies of 2.45 GHz approximately, in order to recover analytes from solids [12,169]. The extraction process is a result of the synergistic combination of bipolar rotation and ionic conduction [169]. Bipolar rotation happens to solvent’s and matrix’s molecules that have a dipole moment when applying electric field, disrupting weak hydrogen bonds [169]. Those phenomena cause the release of thermal energy, increasing the temperature of the solution. Optimal results can be achieved using solvents with higher dielectric constants [169]. High extraction yields for natural matrices can be obtained due to the effect that an electric field has on cell structure [170]. Namely, the traces of water that exist inside the dried material evaporate, increasing intracellular pressure and, thus, creating ruptures in the cell wall [171]. Esquivel-Hernandez et al. extracted 2.46 μg/g tocopherols and 629 μg/g total carotenoids from *A. platensis* using a mixture of methanol, ethyl acetate and light petroleum (1:1:1 *v*/*v*/*v*) at 50 °C [30].

## 6. Conclusions

An in-depth investigation of the literature on the field of SFE application for the recovery of valuable extracts from microalgae has been performed and presented in comprehensive and easily read Tables. SFE using CO_2_ as solvent is suitable for the extraction of solvent-free, high-quality products that, due to the low to moderate operating temperatures applied, maintain their bioactive properties.

A total of thirty-eight different microalgae species are included in this study, and SFE operating conditions are presented along with the extracts’ yield, bioactive compounds content and properties. Modeling attempts of the extraction process are also reported as such information is important for the optimization and scale-up of the process. Finally, other extraction methods—if available—are briefly presented for comparison purposes.

*Arthrospira* (*Spirulina*), *Chlorella*, *Dunaliella*, *Haematococcus* and *Nannochloropsis* are the most investigated microalgae in the literature regarding SFE, which results in promising extracts for applications in either food and cosmetics or biofuels industries.

## Figures and Tables

**Table 1:** Microalgal species and literature data mentioned in this study.

Algae	Pretreatment ^1^	Carotenoids ^1^	Chlorophylls ^1^	Other Bioactive ^1^	Lipids ^1^	Fatty Acids ^1^	TPC AO AM ^1,2^	Kinetic Model ^3^	Exp. Design ^3^	Other Methods ^3^	Ref.
*Arthrospira* *maxima*						✓				✓	[24]
	✓					✓		✓		✓	[25,26]
	✓	✓				✓		✓	✓		[27]
	✓	✓									[28]
*Arthrospira* *pacifica*		✓							✓	✓	[29]
*Arthrospira* *platensis*	✓	✓				✓			✓	✓	[30]
						✓			✓	✓	[31]
	✓					✓			✓	✓	[32]
					✓	✓					[33]
	✓	✓	✓				AO				[34]
		✓		✓		✓			✓		[35]
						✓	AO AM				[36]
			✓						✓		[37]
	✓							✓			[38]
	✓					✓		✓		✓	[39]
*Botryococcus* *braunii*				✓						✓	[24,40]
						✓				✓	[41]
*Chaetoceros muelleri*							AM				[42]
*Chlorella* *protothecoides*								✓		✓	[43]
	✓					✓		✓			[44]
	✓										[45]
*Chlorella* *pyrenoidosa*							AO		✓		[46]
	‘✓	✓									[47]
*Chlorella* *saccharophila*						✓			✓		[48]
*Chlorella* *sorokiniana*	✓	✓	✓					✓		✓	[49]
*Chlorella* sp.		✓	✓		✓						[50]
									✓		[51]
	✓	✓	✓							✓	[52]
					✓						[53]
					✓						[54]
*Chlorella* *vulgaris*								✓	✓	✓	[55]
										✓	[56]
	✓					✓			✓		[57]
	✓	✓								✓	[58]
		✓	✓							✓	[59]
	✓				✓						[60]
	✓	✓									[24]
	✓	✓								✓	[61]
	✓				✓						[62]
	✓							✓			[38]
	✓				✓					✓	[63]
	✓	✓								✓	[40]
	✓	✓									[64]
	✓						TPC			✓	[65]
		✓	✓				AO TPC	✓	✓	✓	[66]
						✓					[67]
*Chlorococcum* sp.	✓					✓		✓		✓	[68]
*Chlorococcum* *littorale*	✓	✓	✓								[69]
Commercial DHA algae	✓									✓	[70]
*Crypthecodinium cohnii*	✓					✓				✓	[71]
*Cylindrotheca. closterium*	✓							✓			[38]
*Dunaliella* *salina*	✓	✓					AO		✓		[72]
	✓								✓		[73]
	✓	✓	✓						✓	✓	[74]
	✓							✓			[75]
	✓	✓	✓						✓	✓	[76]
	✓	✓	✓						✓	✓	[77]
	✓	✓				✓					[78]
		✓									[79]
*Haematococcus* *pluvialis*		✓							✓		[80]
	✓	✓					AO		✓	✓	[81]
	✓	✓						✓	✓		[82]
	✓	✓							✓	✓	[83]
	✓	✓								✓	[84]
	✓	✓			✓					✓	[85]
	✓	✓						✓			[28]
	✓	✓								✓	[86]
	✓	✓								✓	[87]
	✓	✓								✓	[88]
	✓	✓									[89]
		✓					AO		✓	✓	[90]
	✓	✓				✓					[91]
	✓	✓									[92]
*Isochrysis* sp.	✓				✓	✓		✓		✓	[93]
*Isochrysis* *galbana*	✓	✓	✓						✓	✓	[94]
*Monoraphidium* sp.	✓	✓	✓							✓	[95]
*Nannochloropsis* *gaditana*	✓	✓	✓						✓	✓	[96]
	✓	✓	✓						✓	✓	[76]
	✓	✓						✓			[75]
	✓					✓	AO	✓	✓	✓	[93]
	✓	✓						✓		✓	[97]
	✓					✓	AO				[98]
*Nannochloropsis* *granulata*	✓					✓				✓	[99]
	✓			✓	✓						[100]
*Nannochloropsis* *oculata*	✓	✓					AO			✓	[101]
	✓				✓	✓				✓	[102]
	✓			✓		✓				✓	[103]
	✓			✓	✓	✓		✓			[38]
*Nannochloropsis* *salina*								✓			[44]
*Nannochloropsis* sp.	✓					✓		✓		✓	[104]
	✓	✓	✓		✓					✓	[105]
	✓				✓	✓				✓	[106]
*Ochromonas danica*	✓				✓						[107]
*Pavlova* sp.	✓					✓				✓	[108]
*Phaeodactylum* *tricornutum*	✓				✓	✓				✓	[109]
*Phormidium* *valderianum*		✓		✓			AO TPC		✓	✓	[110]
*Scenedesmus* *almeriansis*	✓	✓							✓	✓	[111]
	✓				✓	✓		✓		✓	[93]
	✓	✓			✓	✓				✓	[112]
*Scenedesmus* *dimorphus*	✓					✓				✓	[113]
*Scenedesmus* *obliquus*	✓				✓	✓		✓		✓	[44]
	✓				✓	✓				✓	[2]
	✓	✓	✓						✓	✓	[114]
	✓				✓						[115]
	✓	✓	✓							✓	[116]
*Scenedesmus* *obtusiusculus*	✓					✓				✓	[2]
*Scenedesmus* sp.				✓							[117]
	✓	✓								✓	[118]
	✓	✓								✓	[119]
	✓				✓				✓	✓	[120]
	✓				✓					✓	[121]
*Skeletonema* *costatum*	✓										[107]
*Synechococcus* sp.	✓	✓							✓	✓	[122]
	✓	✓	✓							✓	[76]
		✓	✓						✓	✓	[123]
	✓							✓			[75]
	✓	✓				✓				✓	[17]
*Tetraselmis* *chui*	✓									✓	[124]
*Tetraselmis* sp.	✓				✓			✓		✓	[93]
	✓				✓					✓	[125]

^1^ The data of these columns are analytically presented in Table 2 (pages 8–23), ^2^ Total Phenolic Content (TPC), Antioxidant Activity (AO) or Antimicrobial Activity (AM), ^3^ The data of these columns are analytically presented in Table 3 (pages 24–39).

**Table 2:** SFE conditions applied to microalgae and extracts’ properties and composition.

Algae	Pretreatment	Parametric Investigation	Optimal Conditions	Ext. Yield/Recovery	Carotenoids	Chlorophylls	Other Pigments	Extract Properties	Lipids	Fatty Acids	Ref.
*A. maxima*	GR	T (20–70 °C), P (15–18 MPa), CO_2_ Flow (3.33 × 10^−5^ kg/s), t (660 min)	T (30 °C), P (18 MPa), CO_2_ Flow (3.33 × 10^−5^ kg/s) t (660 min)		2.27 mg T.CAR/0.8 kg/cm^3^ bed					23.64 mg/0.8 kg/cm^3^ bed FA content	[27]
	Crushed by cutting mills	T (60 °C), P (30 MPa), Co-solv (EtOH 0–10% *w*/*w*)	T (60 °C), P (30 MPa), Co-solv (EtOH 10% *w*/*w*)	2.97%	3% PHY >97% AST Rec						[28]
		T (50–60 °C), P (25–35 MPa), Co-solv (EtOH 0–10% *v*/*v*)	T (60 °C), P (35 MPa), Co-solv (EtOH 10% *v*/*v*)							0.44% GLA	[24]
	LY and GR	T (50–60 °C), P (25–35 MPa), CO_2_ Flow (2 g/min), t (390 min), Co-solv (EtOH 0–10% *v*/*v*)	T (60 °C), P (35 MPa), CO_2_ Flow (2 g/min), t (390 min), Co-solv (EtOH 10% *v*/*v*)							0.44% GLA	[25,26]
*A. pacifica*		T (40–80 °C), P (15–35 MPa), CO_2_ Flow (2 mL/min), t (40–100 min), Co-solv (EtOH 5–15% *v*/*v*)	T (60–80 °C), P (35 MPa), CO_2_ Flow (2 mL/min), t (100 min), Co-solv (EtOH 15% *v*/*v*)		48 mg/100 g ZEA, 7.5 mg/100 g β-CRY 118 mg/100 g β-CAR						[29]
*A. platensis*	LY and milled	T (45–60 °C), P (15–45 MPa), CO_2_ Flow (0.015 kg/h), t (50 min), Co-solv (EtOH 26.70–53.22% *v*/*v*)	T (60 °C), P (45 MPa), CO_2_ Flow (0.015 kg/h), t (50 min), Co-solv (EtOH 53.22% *v*/*v*)	4.07%	283 μg/g T.CAR		5.01 μg/g TOC			34.76 mg/g FA	[30]
		T (32–48 °C), P (20–40 MPa), t (120–240 min), Co-solv (EtOH)	T (48 °C), P (20 MPa), t (240 min), Co-solv (EtOH)	10.26 g/kg	77.8 g/kg β-CAR 113.2 g/kg Vitamin A		85.1 g/kg Flavonoids 3.4 g/kg α-TOC			35.32% PA, 21.66% LNA, 20.58% LOA	[35]
	Air-dried	T (55 °C), P (8–36 MPa), CO_2_ Flow (3 L/h), Co-solv (EtOH 10% mol)	T (55 °C), P (22 MPa), CO_2_ Flow (3 L/h), Co-solv (EtOH 10% mol)	0.63% (SEP 1) 2.46% (SEP 2)	178.2 ppm ZEA (SEP 1) 109.3 ppm ZEA (SEP 2) 19.8 ppm MYX fucoside (SEP 1) 52.9 ppm MYX fucoside (SEP 2) 55.0 ppm β-CAR (SEP 2)	CHL-a 480.1 ppm (SEP 1) 55.0 ppm (SEP 2)		AO 66.6 μg/mL EC_50_ (SEP 1) 73.5 μg/mL EC_50_ (SEP 2)			[34]
		T (40–80 °C), P (10–30 MPa), t (30–90 min), Co-solv (EtOH 10–50% *v*/*v*)	T (40 °C), P (30 MPa), t (90 min), Co-solv (EtOH 50% *v*/*v*)	6.7% *w*/*w*						24.7% GLA Rec	[31]
		T (33.18–66.82 °C), P (23.2–56.8 MPa), CO_2_ Flow (0.24–0.9 kg/h), t (0–120 min soaking and 30–180 min extraction) Co-solv (MeOH, ACE, EtA 0–10 mL, Aq.EtOH (20–80%) 5–28.4 mL)	T (53.4 °C), P (48.7 MPa), CO_2_ Flow (0.6 kg/h), t (60 min soaking and 120 min extraction) Co-solv (Aq.EtOH (40%) 21.2 mL)			6.84 mg/g CHL-a					[37]
	LY	T (40 °C), P (31.6–48.4 MPa), CO_2_ Flow (0.7 L/min), t (26.4–94 min), Co-solv (EtOH 9.64–16.36 mL)	T (40 °C), P (40 MPa), CO_2_ Flow (0.7 L/min), t (60 min), Co-solv (EtOH 13.7 mL)							102% GLA Rec	[32]
	Air-flow dried	T (60 °C), P (40 MPa), CO_2_ Flow (0.35 kg/h)	T (60 °C), P (40 MPa), CO_2_ Flow (0.35 kg/h)	10.98%							[38]
	LY and GR	T (40–55 °C), P (25–70 MPa), CO_2_ Flow (10 kg/h), t (90–240 min)	T (55 °C), P (70 MPa), CO_2_ Flow (10 kg/h), t (90 min)	7.79% Lipid						37–41% Total FA	[39]
		T (27–83 °C), P (7.8–36.1 MPa), t (75 min), Co-solv (EtOH 0–10% *v*/*v*)	T (55 °C), P (22–32 MPa), t (75 min), Co-solv (wihout) or T (75 °C), P (32 MPa), t (75 min) Co-solv (EtOH 10% *v*/*v*)					AO (EC_50_) 66.7 μg/mL (SEP 1) 36.1 μg/mL (SEP 2) OR 20.0 μg/mL (SEP 1), 129.4 μg/mL (SEP 2) MBC 10–30 mg/mL *E. coli*, 10–25 mg/mL *S.aureus*, 10–15 mg/mL *C.albicans*, >35 mg/mL *A.niger*		44.4%(SEP 1), 36.6% (SEP 2) PA, 30.6%(SEP 1), 25%(SEP 2) OA	[36]
		T (40 °C), P (30–40 MPa), CO_2_ Flow (24 kg/h), t (120–240 min)	T (40 °C), P (35 MPa), CO_2_ Flow (24 kg/h), t (240 min)	7.2% lipid						Composition 16.91% OA, 36.51% LA., 9.16% α-LNA., 19.68% GLA.	[33]
*Botryococcus* *braunii*		T (40 °C), P (12.5–30 MPa)	T (40 °C), P (30 MPa)				~72 g/kg Hydrocarbons				[24,40]
		T (50–80 °C), P (20–25 MPa), t (10–150 min)	T (50 °C), P (25 MPa)	~10.5%						~18% FA	[41]
*Chaetoceros muelleri*		T (40–80 °C), P (20–40 MPa), t (60 min), Co-solv (EtOH 0.2 mL)	T (40 °C), P (40 MPa), t (60 min), Co-solv (EtOH 0.2 mL)	3.9%				MBC 12 mg/mL *E. coli* 12 mg/mL *S. aureus* 7 mg/mL *C. albicans*			[42]
*Chlorella* *protothecoides*		T (50 °C), P (35 MPa), CO_2_ Flow (0.0439 kg/h), t (180 min)	T (50 °C), P (35 MPa), CO_2_ Flow (0.0439 kg/h), t (180 min)	0.23 g/g_biom_ lipid 75% Rec							[43]
	Oven dried, GR and sieved	T (60 °C), P (30 MPa), CO_2_ Flow (30 g/h), t (90 min), Co-solv (EtOH 5%)	T (60 °C), P (30 MPa), CO_2_ Flow (30 g/h), t (90 min), Co-solv (EtOH 5%)	10% Lipid						Composition 25.68% SFA 13.1% MUFA 61.77% PUFA 15.13% Ω-3 23.63% Ω-6	[44]
	Oven dried, milled, MW, sonication, autoclave	T (35–70 °C), P (15–30 MPa), CO_2_ Flow (3–7 g/min)	T (70 °C), P (30 MPa), CO_2_ Flow (3 g/min)	21%							[45]
*C. pyrenoidosa*	LY, superfine pulverized	T (40–60 °C), P (20–30 MPa), CO_2_ Flow (20 kg/h), t (2–8 h), Co-solv (EtOH 0–70%)	T (50 °C), P (25 MPa), CO_2_ Flow (20 kg/h), t (4 h), Co-solv (EtOH 50%)		87% LUT Rec						[47]
		T (32–55 °C), P (25–40 MPa), CO_2_ Flow (15–30 kg/h), t (1.5–180 min), Co-solv (EtOH 0–1.5 mL/g_biom_)	T (32 °C), P (40 MPa), CO_2_ Flow (20 kg/h), t (180 min), Co-solv (EtOH 1 mL/g_biom_)	7.78%				AO 42.03% Inhibition			[46]
*C. saccharophila*		T (42–73 °C), P (24.1–41.4 MPa), t (30–90 min)	T (73 °C), P (24.1 MPa), t (86 min)							20.4% T-FAME Comp.	[48]
*C. sorokiniana*	High-pressure cell disruption	T (40–60 °C), P (10–30 MPa), t (180 min), Co-solv (EtOH 0–10%)	T (50 °C), P (20 MPa), t (180 min), Co-solv (EtOH 5%)	35.03 mg/g	0.526 mg/g (18.8% Rec) LUT 0.056 mg/g (26.2% Rec) VIO 0.051 mg/g (16.8% Rec) ZEA 0.557 mg/g (73.7% Rec) Carotene	4.60 mg/g (36.2% Rec) CHL-a 3.92 mg/g (82.3% Rec) CHL-b					[49]
*Chlorella* sp.		T (40–60 °C) P (15–30 MPa), CO_2_ Flow (15 g/min), t (180 min), Co-solv (Hexane/MeOH 1–3 *v*/*v*)	T (40 °C), P (30 MPa), CO_2_ Flow (15 g/min), t (180 min), Co-solv (Hexane/MeOH 2 *v*/*v*)	47.2%							[51]
		T (60 °C), P (20 MPa), CO_2_ Flow (0.5–2 L/min), t (240 min), Co-solv (Hexane 0.4 mL/min)	T (60 °C), P (20 MPa), CO_2_ Flow (0.5 L/min), t (240 min), Co-solv (Hexane 0.4 mL/min)						63.78% Lipid Y		[53]
		T (40–60 °C), P (20–30 MPa), CO_2_ Flow (6.7–20 g/min)	T (60 °C), P (30 MPa),	2.2%					79.53% Lipid Y		[54]
		T (40–50 °C), P (15–30 MPa), CO_2_ Flow (0.5–4 g/min), Co-solv (EtOH 0–30%)	T (40 °C), P (30 MPa), CO_2_ Flow (1.88 g/min), Co-solv (EtOH ~30%)		160–222 μg/g T.CAR	830–1050 μg/g CHL-a			360–400 μg/g Ergosterol		[50]
	LY and bead milled	T (60 °C), P (20–30 MPa), CO_2_ Flow (30 g/h), t (180 min), Co-solv (EtOH 0–5%)	T (60 °C), P (30 MPa), CO_2_ Flow (30 g/h), t (180 min), Co-solv (EtOH 5% *w*/*v*)		5 mg/g (26.2% Rec) T.CAR	9 mg/g T. CHL					[52]
*C. vulgaris*		T (60–80 °C), P (20–50 MPa), CO_2_ Flow (2.5 mL/min), t (3–6 h), Co-solv (EtOH or ACE 7.5% *v*/*v*)	T (60 °C), P (30 MPa), CO_2_ Flow (2.5 mL/min), t (6 h), Co-solv (EtOH 7.5% *v*/*v*)		3 mg/g LUT 0.06 mg/g Carotene	7 mg/g CHL-a 3 mg/g CHL-b					[59]
	LY, 3 degrees of crushing	T (40–55 °C), P (20–35 MPa)	T (55 °C), P (35 MPa)		40% T.CAR Rec						[24]
	3 degrees of crushing	T (40 °C), P (12.5–30 MPa), CO_2_ Flow (0.04 kg/h)	T (40 °C), P (30 MPa), CO_2_ Flow (0.04 kg/h)		>70% T.CAR Rec						[40]
	MW	T (40–70 °C), P (20–28 MPa), CO_2_ Flow (10 kg/h), t (9 h)	T (70 °C), P (28 MPa), CO_2_ Flow (10 kg/h), t (9 h)	4.86%						26.598 mg/100 mg_oil_ PA 27.296 mg/100 mg_oil_ OA 10.403 mg/100 mg_oil_ LNA 16.163 mg/100 mg_oil_ a-LNA	[57]
	Air dried	T (45 °C), P (45 MPa), CO_2_ Flow (25 g/min)	T (45 °C), P (45 MPa), CO_2_ Flow (25 g/min)	~14 %							[38]
	LY, crushed	T (40–55 °C), P (15–35 MPa), CO_2_ Flow (0.4 dm^3^/min), t (125–480)	T (55 °C), P (35 MPa), CO_2_ Flow (0.4 dm^3^/min), t (330 min)		5% T.CAR						[61]
		T (40–60 °C), P (11–25 MPa), CO_2_ Flow (20–40 g/min)	T (60 °C), P (25 MPa), CO_2_ Flow (40 g/min)	3.37%	21.14 mg/g_extr_ T.CAR 10.00 mg/g_extr_ Sel. CAR	35.55 mg/g_extr_ T.CHL		AO 44.35 mg_extr_/mg_DPPH_ TPC 18.29 mg_GA_/g_extr_			[66]
	LY	T (50 °C), P (31 MPa), CO_2_ Flow (6 NL/min), t (20 min), Co-solv (Aq. EtOH (50%) 50 mL)	T (50 °C), P (31 MPa), CO_2_ Flow (6 NL/min), t (20 min), Co-solv (Aq. EtOH (50%) 50 mL)	8.71%				TPC 13.40 mg GAE/g_extr_			[65]
		T (40–60 °C), P (27.6–48.3 MPa), CO_2_ Flow (1–3 g/min), t (1–180 min)	T (60 °C), P (48.3 MPa), CO_2_ Flow (3 g/min), t (180 min)	17.7%							[55]
		T (40–80 °C), P (27.6–62.1 MPa), t (180 min)	T (80 °C), P (62.1 MPa), t (180 min)	19% >99% Rec							[56]
	Crushed (3 degrees)	T (40 °C), P (30 MPa), CO_2_ Flow (0.34–0.6 L/min), Co-solv (EtOH or oil)	T (40 °C), P (30 MPa), CO_2_ Flow (0.34 L/min), Co-solv (oil)								[58]
	LY, crushed	T (40–55 °C), P (20–35 MPa), CO_2_ Flow (0.4 dm^3^/min), t (125–480 min)	T (55 °C), P (35 MPa), CO_2_ Flow (0.4 dm^3^/min), t (330 min)	0.05%					54.26 mg/g Total Lipid Y		[62]
		T (40–80 °C), P (20–37 MPa), CO_2_ Flow (100–200 g/min), t (60 min), Co-solv (Hexane/EtOH (1:1) 4–12 *w*/*w* biomass)	T (40 °C), P (37 MPa)							Composition 30.05% PA 30.22% STA 3.24% LAA 4.82% MA 3.01% AA 2.54% PLA 3.38% OA 1.63% LNA 1.71% DHA 2.98% EPA	[67]
	LY	T (50 °C), P (25 MPa), CO_2_ Flow (0.5 kg/h), t (210–230 min) Co-solv (EtOH 0–10% *v*/*v*)	T (50 °C), P (25 MPa), CO_2_ Flow (0.5 kg/h), t (230 min), Co-solv (EtOH 10% *v*/*v*)	~40%					97% Rec Neutral Lipid ~25% Rec Glycolipid ~35% Rec Phospholipid		[63]
	Spray-dried, eluent pretreated	T (40–80 °C), P (20–40 MPa), CO_2_ Flow (3 mL/min), t (100 min), Co-solv (EtOH 0.3–0.5 mL/min)	T (40 °C), P (40 MPa), CO_2_ Flow (3 mL/min), t (100 min), Co-solv (EtOH 0.4 mL/min)	~1.8%	52.9% LUT Rec						[64]
*Chlorococcum littorale*	LY	T (60 °C), P (30 MPa), CO_2_ Flow (0.36 dm^3^/min), t (80 min), Co-solv (EtOH 0–10% mol)		~0.2 mg/mg Rec	~89% T.CAR	~48% T.CHL					[69]
*Chlorococcum* sp.	Dried, GR or wet biomass	T (60–80 °C), P (30 MPa), CO_2_ Flow (400 mL/min), t (80 min)		7.1% Lipid						1.4% FAME	[68]
Commercial DHA algae	Lyophilized or high-pressure ruptured	T (30–60 °C), P (10.5–30 MPa), CO_2_ Flow (20 mL/min), t (90–2700 min), Co-solv (EtOH, EtA, 1-Propanol 30:1–10:1)	T (30 °C), P (30 MPa), CO_2_ Flow (20 mL/min), t (2700 min), Co-solv (1-Propanol 30:1)	90.56%							[70]
*Crypthecodinium cohnii*	LY	T (40–50 °C), P (20–30 MPa), CO_2_ Flow (0.6 kg/h), t (180 min)		8.6% Lipid						72% DHA Composition	[71]
*Cylindrotheca closterium*	Air-dried or LY	T (60 °C), P (40 MPa), CO_2_ Flow (0.41 kg/h)		12.73%							[38]
*Dunaliella salina*	LY, homogenized	T (40–60 °C), P (10–50 MPa), CO_2_ Flow (4.5 mmol/min), t (180 min), Co-solv (EtOH 0–5% mol)	T (60 °C), P (40 MPa), CO_2_ Flow (4.5 mmol/min) t (180 min), Co-solv (EtOH 5% mol)	1.2%							[75]
	LY	T (9.8–45.2 °C), P (18.5–44.2 MPa), t (100 min)	T (9.8 °C), P (31.4 MPa), t (100 min)					MBC 3.1 mg/mL *E. coli*, 3.9 mg/mL *S. aureus* MFC 8.3 mg/mL *C. albicans*, 30 mg/mL *A. niger*			[73]
	LY	T (9.8–45.2 °C), P (18.5–44.2 MPa), t (100 min)	T (27.5 °C), P (44.2 MPa), t (100 min)	6.58%	7.199 mg T.CAR/100 mg_extr_, 3.751 mg β-CAR/100 mg_extr_			AO 0.452 mmol TE/g_extr_			[72]
	LY	T (40–60 °C), P (10–50 MPa), CO_2_ Flow (4.5 mmol/min), t (180 min)	T (60 °C), P (40 MPa), CO_2_ Flow (4.5 mmol/min), t (180 min) OR T (60 °C), P (50 MPa), CO_2_ Flow (4.5 mmol/min), t (180 min)		12.17 μg/mg or 9.3 μg/mg T.CAR	0.227 μg/mg or 0.376 μg/mg T.CHL					[74]
	LY	T (40–60 °C), P (10–50 MPa), CO_2_ Flow (4.5 mmol/min), t (180 min), Co-solv (EtOH 5% mol)	T (60 °C), P (40 MPa), CO_2_ Flow (4.5 mmol/min), t (180 min), Co-solv (EtOH 5% mol)		9.629 μg/mg T.CAR	0.700 μg/mg T.CHL					[76]
	Spray-dried	T (30–60 °C), P (10–50 MPa), CO_2_ Flow (3 L/min), t (90 min)	T (55 °C), P (40 MPa), CO_2_ Flow (3 L/min), t (90 min)		115.44 μg/g T.CAR	32.68 μg/g T.CHL					[77]
	GR (in different conditions)	T (50–75 °C), P (10–55 MPa), CO_2_ Flow (7.24–14.48 g/min), t (30–110 min)	T (65 °C), P (14 MPa), CO_2_ Flow (14.48 g/min), t (110 min) OR T (75 °C), P (55 MPa), CO_2_ Flow (14.48 g/min), t (110 min)		25.48% β-CAR Rec				7.91 mg/g OR 8.47 mg/g Lipids	95.88% OR 97.07% FAME Rec	[78]
		T (35–55 °C), P (20–30 MPa), t (180 min), Co-solv (EtOH/MeOH 0–5% *w*/*w*)	T (45 °C), P (20 MPa), t (180 min), Co-solv (EtOH 5% *w*/*w*)		25 g/kg T.CAR						[79]
*Haematococcus pluvialis*		T (40–80 °C), P (30–50 MPa), t (60–240 min)	T (90 °C), P (64.0 MPa), t (174 min)		22.66 mg/g AST						[92]
	Dried	T (40–80 °C), P (30–50 MPa), CO_2_ Flow (3 mL/min), t (60–240 min)	T (80 °C), P (50 MPa), CO_2_ Flow (3 mL/min), t (60 min)		22.844 mg/g (83.05% Rec) OR 11.780 mg/g AST			AO (IC_50_) 2.37 mg/L OR 1.77 mg/L			[81]
	Disrupted	T (40–70 °C), P (30–55 MPa), t (300 min), Co-solv (EtOH 0–8% *v*/*v*)	T (40 °C), P (55 MPa), t (300 min), Co-solv (EtOH 4.5% *v*/*v*)		84% AST Rec						[82]
	LY	T (30–80 °C), P (6.9–34.5 MPa), CO_2_ Flow (2–12 ΝL/min) t (20–100 min), Co-solv (EtOH/H_2_O 19.5–78 mL 0–99.5% *v*/*v*)	T (50 °C), P (31 MPa), CO_2_ Flow (6 ΝL/min) t (20 min), Co-solv (EtOH/H_2_O 9.23 mL/g 99.5% *v*/*v*)		10.92 mg/L (73.9% Rec) AST						[83]
	Dried	T (35–75 °C), P (30–50 MPa), CO_2_ Flow (10 L/h), t (210 min), Co-solv (EtOH 0.5–3.5 mL/g)	T (65 °C), P (43.5 MPa), CO_2_ Flow (10 L/h), t (210 min), Co-solv (EtOH 2.3 mL/g)		87.42% AST						[89]
	LY	T (45 °C), P (11.7–48.3 MPa), CO_2_ Flow (2.7 mL/min) t (240 min)	T (45 °C), P (48.3 MPa), CO_2_ Flow (2.7 mL/min), t (240 min)		84.8% AST Rec				85.3% Total TAG Rec		[85]
	Crushed and/or GR	T (60 °C), P (30 MPa), Co-solv (EtOH 0–9.4% *w*/*w*)	T (60 °C), P (30 MPa), Co-solv (EtOH 9.4% *w*/*w*)		~1.6% AST ~3% PHY						[28]
	LY, crushed (3 degrees)	T (40–60 °C), P (20–30 MPa), Co-solv (EtOH 0–10%)	T (60 °C), P (30 MPa), Co-solv (EtOH 10%)		~59–92% T.CAR Rec, ~76% β-CAR Rec, ~90% AST Rec						[86]
	Dried	T (40–80 °C), P (20–55 MPa), CO_2_ Flow (2–4 mL/min), t (240 min), Co-solv (EtOH 0–7.5% *v*/*v*)	T (70 °C), P (40 MPa), CO_2_ Flow (3 mL/min), t (240 min), Co-solv (EtOH 5% *v*/*v*)		80.6% AST Rec						[87]
	Dried	T (50–80 °C), P (30–50 MPa), CO_2_ Flow (2–4 mL/min), t (300 min), Co-solv (EtOH/Soy bean oil/Olive oil 0–12% *v*/*v*)	T (70 °C), P (40 MPa), CO_2_ Flow (3 mL/min), t (300 min), Co-solv (Olive oil 10% *v*/*v*)		51% AST						[88]
	Disrupted, powdered or homogenized with water	T (40–70 °C), P (35–75 MPa), CO_2_ Flow (10 g/min) t (270–600 min)	T (70 °C), P (55 MPa), CO_2_ Flow (10 g/min) t (270 min) for powdered OR T (70 °C), P (45 MPa), CO_2_ Flow (10 g/min) t (600 min) for homogenized		61% OR 54% AST Rec						[84]
		T (40–70 °C), P (20–35 MPa), CO_2_ Flow (0.06 g/min), t (120 min), Co-solv (EtOH 0–13% *w*/*w*)	T (55 °C), P (20 MPa), CO_2_ Flow (0.06 g/min), t (120 min), Co-solv (EtOH 13% *w*/*w*)	282.5 mg/g	53.48 mg/g (82.3% Rec) AST			AO 0.243 mM TE/g			[90]
	Ball-milled, HPR (H. Red Phase)	T (50–80 °C), P (10–55 MPa), CO_2_ Flow (3.62–14.48 g/min), t (20–120 min)	T (50 or 65 °C), P (55 MPa), CO_2_ Flow (3.62 g/min), t (120 min)	237.4 mg/g	19.72 mg/g (98.6% Rec) AST 4.03 mg/g (52.3% Rec) LUT					21.41 mg/g Y, 93.2% Rec	[91]
	HPR (H. Red Phase)	T (50–80 °C), P (10–55 MPa), CO_2_ Flow (3.62 g/min), t (20–80 min), Co-solv (EtOH 0–1 mL/min)	T (65 °C), P (55 MPa), CO_2_ Flow (3.62 g/min), t (80 min), Co-solv (EtOH 1 mL/min)	280.78 mg/g	18.5 mg/g (~92% Rec) AST 7.15 mg/g (~93% Rec) LUT						[92]
*Isochrysis* *galbana*	LY	T (40–60 °C), P (10–30 MPa), CO_2_ Flow (5 L/min), t (60 min)	T (50 °C), P (30 MPa), CO_2_ Flow (5 L/min), t (60 min)	5%	16.2 mg/g T.CAR	4.5 mg/g T.CHL					[94]
*Isochrysis* sp.	LY and/or MW	T (45 °C), P (30 MPa), CO_2_ Flow (0.4 kg/h), t (120 min) Co-solv (EtOH 5%)		15.5%					9.3% Lipid Y	61.9% Free FA Conversion	[93]
*Monoraphidium* sp.	LY	T (30–60 °C), P (20 MPa), t (15–60 min), Co-solv (EtOH 0–20 mL)	T (60 °C), P (20 MPa), t (60 min), Co-solv (EtOH 20 mL)		2.46 mg/g (101% Rec) AST	29.5 mg/g (103% Rec) T. CHL					[95]
*Nannochloropsis gaditana*	LY, homogenized	T (40–60 °C), P (10–50 MPa), CO_2_ Flow (4.5 mmol/min), t (180 min)	T (60 °C), P (40 MPa), CO_2_ Flow (4.5 mmol/min), t (180 min) OR T (60 °C), P (20 MPa), CO_2_ Flow (4.5 mmol/min), t (180 min)		0.343 μg/mg OR 0.125 μg/mg T.CAR	2.238 μg/mg OR 0.090 μg/mg CHL-a					[96]
	LY	T (40–60 °C), P (20–50 MPa), CO_2_ Flow (4.5 mmol/min), t (180 min) Co-solv (EtOH 5% mol)	T (60 °C), P (50 MPa), CO_2_ Flow (4.5 mmol/min), t (180 min) Co-solv (EtOH 5% mol)		2.893 μg/mg T.CAR	0.369 μg/mg CHL-a					[76]
	LY, ASE	T (50–65 °C), P (25–55 MPa), CO_2_ Flow (7.24–14.48 g/min), t (100 min)	T (65 °C), P (25 MPa), CO_2_ Flow (7.24 - 14.48 g/min), t (100 min)	77.68 mg/g					34.15 mg/g Lipid Y	~7.5 mg/g SFAs, ~8 mg/g MUFAs, ~10.5 mg/g PUFAs ~11.50 mg/g EPA	[98]
	LY and/or MW	T (45 °C), P (30 MPa), CO_2_ Flow (0.4 kg/h), t (120 min) Co-solv (EtOH 5%)		12.9%					7.9% Lipid Y	61.2% Free FA Conversion	[93]
	LY	T (40–60 °C), P (20–50 MPa), CO_2_ Flow (4.5 mmol/min), t (180 min) Co-solv (EtOH 0–5% mol)	T (60 °C), P (50 MPa), CO_2_ Flow (4.5 mmol/min) t (180 min) Co-solv (EtOH 5% mol)		~0.33% T.CAR						[75]
	LY, High-pressure homogenized	T (55 °C), P (40 MPa), CO_2_ Flow (10 L/min), t (270 min)		11.48%	0.18 mg/g (8.3% Rec) VIO						[97]
*N. granulata*	LY, milled	T (50–90 °C), P (35–55 MPa), CO_2_ Flow (100 g/min), t (180–270 min)	T (70 °C), P (35 MPa), CO_2_ Flow (100 g/min), t (270 min)	28.45 mg/g ash free biomass						18.23 mg/g FAME	[99]
	LY	T (70–90 °C), P (35 MPa), CO_2_ Flow (100 g/min), t (270 min)	T (70 °C), P (35 MPa), CO_2_ Flow (100 g/min), t (270 min)				165.9 g/kg Carbohydrates, 363.9 g/kg Sum of amino acids, 21.9 g/kg Non-protein		256.3 g/kg Crude Lipid		[100]
*N. oculata*	LY, GR	T (50 °C), P (25–35 MPa), CO_2_ Flow (20 mL/min), Co-solv (EtOH, DCM, Toluene, n-Hexane)	T (50 °C), P (35 MPa), CO_2_ Flow (20 mL/min), Co-solv (EtOH)		13.7 mg/g_extr_ (63.2% Rec) ZEA			AO 1.612 mg/mL sample EC_50_, 0.313 mmol TE/g sample			[101]
	LY, homogenized	T (40–80 °C), P (20.7–62.1 MPa), CO_2_ Flow (24 mL/min), t (240 min)	T (40 °C), P (20.7 MPa), CO_2_ Flow (24 mL/min), t (240 min)	47.30 mg/g			10.36 mg/g Total TOC			Composition 35% T. SFA 45.31% T.MUFA 19.69% T.PUFA	[103]
	LY or air dried, crushed or GR	T (60 °C), P (30–85 MPa), CO_2_ Flow (0.5–100 kg/h), t (270 min)	T (60 °C), P (40 MPa), CO_2_ Flow (0.5 kg/h), t (270 min)	~15%					Composition 93.82% Triglycerides 1.80% Sterol	2.62% Free FA Comp.	[102]
	LY or air dried	T (60 °C), P (40 MPa), CO_2_ Flow (0.4–0.5 kg/h), t (120 min)	T (60 °C), P (40 MPa), CO_2_ Flow (0.5 kg/h), t (120 min)	~12%			1.76% Pigments Comp.		Composition 93.82% Triglycerides 1.80% Sterol	2.62% Free FA Comp.	[38]
*N. gaditana*	LY	T (40–60 °C), P (20–50 MPa), CO_2_ Flow (4.5 mmol/min), t (180 min) Co-solv (EtOH 0–5% mol)	T (60 °C), P (50 MPa), CO_2_ Flow (4.5 mmol/min) t (180 min) Co-solv (EtOH 5% mol)		~0.33% T.CAR						[75]
	LY, High-pressure homogenized	T (55 °C), P (40 MPa), CO_2_ Flow (10 L/min), t (270 min)		11.48%	0.18 mg/g (8.3% Rec) VIO						[97]
*N. granulata*	LY, milled	T (50–90 °C), P (35–55 MPa), CO_2_ Flow (100 g/min), t (180–270 min)	T (70 °C), P (35 MPa), CO_2_ Flow (100 g/min), t (270 min)	28.45 mg/g ash free biomass						18.23 mg/g FAME	[99]
	LY	T (70–90 °C), P (35 MPa), CO_2_ Flow (100 g/min), t (270 min)	T (70 °C), P (35 MPa), CO_2_ Flow (100 g/min), t (270 min)				165.9 g/kg Carbohydrates, 363.9 g/kg Sum of amino acids, 21.9 g/kg Non-protein		256.3 g/kg Crude Lipid		[100]
*N. oculata*	LY, GR	T (50 °C), P (25–35 MPa), CO_2_ Flow (20 mL/min), Co-solv (EtOH, DCM, Toluene, n-Hexane)	T (50 °C), P (35 MPa), CO_2_ Flow (20 mL/min), Co-solv (EtOH)		13.7 mg/g_extr_ (63.2% Rec) ZEA			AO 1.612 mg/mL sample EC_50_, 0.313 mmol TE/g sample			[101]
	LY, homogenized	T (40–80 °C), P (20.7–62.1 MPa), CO_2_ Flow (24 mL/min), t (240 min)	T (40 °C), P (20.7 MPa), CO_2_ Flow (24 mL/min), t (240 min)	47.30 mg/g			10.36 mg/g Total TOC			Composition 35% T. SFA 45.31% T.MUFA 19.69% T.PUFA	[103]
	LY or air dried, crushed or GR	T (60 °C), P (30–85 MPa), CO_2_ Flow (0.5–100 kg/h), t (270 min)	T (60 °C), P (40 MPa), CO_2_ Flow (0.5 kg/h), t (270 min)	~15%					Composition 93.82% Triglycerides 1.80% Sterol	2.62% Free FA Comp.	[102]
	LY or air dried	T (60 °C), P (40 MPa), CO_2_ Flow (0.4–0.5 kg/h), t (120 min)	T (60 °C), P (40 MPa), CO_2_ Flow (0.5 kg/h), t (120 min)	~12%			1.76% Pigments Comp.		Composition 93.82% Triglycerides 1.80% Sterol	2.62% Free FA Comp.	[38]
*N. salina*		T (60 °C), P (30 MPa), CO_2_ Flow (0.4 kg/h), t (90 min), Co-solv (EtOH 5%)		~30%							[44]
*Nannochloropsis* sp.	LY, GR	T (40–55 °C), P (40–70 MPa), CO_2_ Flow (10 kg/h), t (360 min)	T (55 °C), P (40 MPa), CO_2_ Flow (10 kg/h), t (360 min)	~257 mg/g Lipid						Composition 25.3% SFA 20.1% Monoenoic 54.6% PUFA 44% n-3 PUFAs	[104]
	Dried, milled	T (40–60 °C), P (12.5–30 MPa), CO_2_ Flow (0.35–0.62 g/min), t (60–105 min), Co-solv (EtOH 0–20% *w*/*w*)	T (40 °C), P (30 MPa), CO_2_ Flow (0.62 g/min), Co-solv (EtOH 20% *w*/*w*)		Composition: 13.71% AST 22.35% LUT, 13.20% VIO/NEO, 34.30% VAU, 4.71% CAN, 5.06% β-CAR		~1 mg/g Pigment Rec		45% Lipid Y		[105]
	Bead milled	T (50–75 °C), P (10–55 MPa), CO_2_ Flow (7.24–14.48 g/min), t (100 min)	T (75 °C), P (55 MPa), CO_2_ Flow (14.48 g/min), t (100 min) OR T (50 °C), P (40 MPa), CO_2_ Flow (14.48 g/min), t (100 min)	94.28 mg/g OR 58.26 mg/g					18.39 mg/g OR 10.37 mg/g Lipid Y	5.69 mg/g (15.59% Rec) EPA OR 0.12 mg/g (79.63% Rec) DHA	[106]
*Ochromonas danica*	LY	T (40 °C), P (17.2–31 MPa), t (~240 min)	T (40 °C), P (17.2 MPa), t (~240 min)						234.2 mg/g Lipid Y		[107]
*Pavlova* sp.	Bead milled	T (45 °C), P (30.6 MPa), t (360 min)		17.9%						15.7% (98.7% Rec) FAME	[108]
*Phaeodactylum* *tricornutum*	MW with DES	T (45 °C), P (30.6 MPa), CO_2_ Flow (2.5 L/min), t (360 min)							7.1% Lipid Y	7.0% TFA Y, 1.0% EPA Y, 2.0% PUFA Y	[109]
*Phormidium* *valderianum*		T (35.86–64.14 °C), P (13.79–56.21 MPa), CO_2_ Flow (2 L/min), t (90 min)	T (50 °C), P (50 MPa), CO_2_ Flow (2 L/min), t (90 min)	3.96 mg/g	13.43 μg β-CAR eq. /g T.CAR		1.41 mg/g Anatoxin-a	2596.57 μg BHT eq./g Reducing Power, 5.29 mM FeSO4 eq./g FRAP value, 0.38 mg/mL IC_50_ TPC 94.87 μg GAE/g			[110]
*Scenedesmus* *almeriansis*	LY, milled, and/or bead milled with alumina A	T (32–60 °C), P (20–60 MPa), CO_2_ Flow (1 g/min), t (300 min)	T (60 °C), P (40 MPa), CO_2_ Flow (1 g/min), t (300 min)		0.0466 mg/g LUT 1.50 mg/g β-CAR						[111]
	LY and matrix solid-phase dispersion	T (50–65 °C), P (25–55 MPa), CO_2_ Flow (7.24–14.48 g/min), t (120 min)	T (65 °C), P (55 MPa), CO_2_ Flow (14.48 g/min), t (120 min)	8.74 mg/g	2.97 mg/g (17% Rec) LUT				3.42 mg/g Lipid Y	15% FA Rec	[112]
	LY and/or MW	T (45 °C), P (30 MPa), CO_2_ Flow (0.4 kg/h), t (90 min), Co-solv (EtOH 5% *v*/*v*)		13.2%					10.1% Lipid Y	76.5% Free FA Conversion	[93]
*S. dimorphus*	LY and/or MW, sonicated and bead milled	T (50–100 °C), P (16.6–50 MPa), t (60 min)	T (100 °C), P (41.4 MPa), t (60 min)							98.8% FAME Rec	[113]
*S. obliquus*	LY and/or high-pressure homogenized	T (40–60 °C), P (10–40 MPa), CO_2_ Flow (7 L/min), t (120 min)	T (50 °C), P (36 MPa), CO_2_ Flow (7 L/min), t (120 min)	0.97%	35.85 mg/g_extr_ T.CAR	11.03 mg/g_extr_ T.CHL					[114]
	LY	T (20–200 °C), P (7–80 MPa), t (540 min)	T (20 °C), P (120 MPa), t (540 min)	6.4%					92% Lipid Rec	59% PUFA Conc.	[2]
	Dried	T (45–65 °C), P (15–30 MPa), CO_2_ Flow (0.4 kg/h), t (30–90 min), Co-solv (EtOH 5% *v*/*v*)	T (60 °C), P (30 MPa), CO_2_ Flow (0.4 kg/h), t (30 min), Co-solv (EtOH 5% *v*/*v*) OR T (65 oC), P (30 MPa), CO_2_ Flow (0. kg/h), t (90 min), Co-solv (EtOH 5% *v*/*v*)	24.67%					18.15% Lipid Y	73.57% Free FA Conv. 33.76% Ω-3, 23.63% Ω-6, 26.71% SFA, 22.00% MUFA, 51.28% PUFA	[44]
	LY, homogenized	T (40–60 °C), P (15–25 MPa), CO_2_ Flow (2–4.3 g/min), t (240 min), Co-solv (EtOH 0–9.5% *v*/*v*)	T (60 °C), P (25 MPa), CO_2_ Flow (2 g/min), t (240 min) Co-solv (EtOH 0% *v*/*v*)		0.182 mg/g T.CAR	0.016 mg/g CHL-a, 0.016 mg/g CHL-b, 0.011 mg/g CHL-c					[116]
	LY, protein concentrate	T (40 °C), P (37.9 MPa), CO_2_ Flow (3 sL/min), Co-solv (EtOH 0–15% *v*/*v*)	T (40 °C), P (37.9 MPa), CO_2_ Flow (3 sL/min), Co-solv (EtOH 15% *v*/*v*)						Composition 12.48% Lipid 67.89% Neutral Lipids 22.52% Glycolipids 9.59% Phospholipids		[115]
*S. obtusiusculus*	LY	T (20 °C), P (12 MPa), t (540 min)		6.4%						42.52% FA Y	[2]
*Scenedesmus* sp.	LY, GR	T (35–65 °C), P (20–50 MPa), CO_2_ Flow (1.38–4.02 g/min)	T (53 °C), P (50 MPa), CO_2_ Flow (1.9 g/min)	7.06%					7.41% Lipid Y		[120]
	LY	T (35–50 °C), P (40 MPa), t (120–360 min), Co-solv (MeOH)	T (35 °C), P (40 MPa) t (360 min), Co-solv (MeOH)						19.32% Lipid Y		[121]
	LY	T (35–80 °C), P (20–40 MPa), CO_2_ Flow (750–800 mL/min), t (60 min), Co-solv (MeOH, EtOH, Propanol, Butanol, ACE 0–40% mol)	T (70 °C), P (40 MPa), CO_2_ Flow (750–800 mL/min) t (60 min), Co-solv (EtOH 30% mol)		2.210 mg/g (76.7% Rec) LUT						[118]
	LY, GR	T (60 °C), P (30 MPa), CO_2_ Flow (2 mL/min), t (60 min), Co-solv (EtOH 0–10% mol)	T (60 °C), P (30 MPa), CO_2_ Flow (2 mL/min), t (60 min), Co-solv (EtOH 10% mol)		72.9 μg/g AST 436.1 μg/g LUT 59.9 μg/g β-CAR 670.8 μg/g NEO 89.6 μg/g ZEA						[119]
		T (40 °C), P (35 MPa), CO_2_ Flow (800 mL/min), t (60 min), Co-solv (MeOH/Water 90:10 *v*/*v*, 0.3 mL)					0.96 ng/g Daidzin, 4.91 ng/g Genistin, 9.14 ng/g Ononin, 10.6 ng/g Daidzein, 3.82 ng/g Sissotrin, 6.11 ng/g Genistein 5.92 ng/g Formononetin, 6.8 ng/g Biochanin A				[117]
*Skeletonema* *costatum*	LY	T (40 °C), P (17.2–31 MPa) t (240 min)	T (40 °C), P (24 MPa) t (240 min)	~65 mg/g							[107]
*Synechococcus* sp.	LY and homogenized	T (40–60 °C), P (20–50 MPa), CO_2_ Flow (4.5 mmol/min), t (180 min), Co-solv (EtOH 0–5% mol)									[75]
		T (40–60 °C), P (20–50 MPa), CO_2_ Flow (4.5 mmol/min), t (180 min)	T (50 °C), P (30 MPa), CO_2_ Flow (4.5 mmol/min) t (180 min)		1.511 μg/mg T.CAR	0.078 μg/mg T.CHL					[123]
	LY	T (40–60 °C), P (20–50 MPa), CO_2_ Flow (4.5 mmol/min), t (180 min), Co-solv (EtOH 5% mol)	T (50 °C), P (30 MPa), CO_2_ Flow (4.5 mmol/min), t (180 min), Co-solv (EtOH 5% mol)		1.86 μg/mg T.CAR	0.286 μg/mg T.CHL					[76]
	LY	T (40–60 °C), P (20–50 MPa), CO_2_ Flow (4.5 mmol/min) t (240 min), Co-solv (EtOH 15% mol)	CO_2_ Flow (4.5 mmol/min) t (240 min), Co-solv (EtOH 15% mol) T (50 °C), P (35.8 MPa), OR T (59 °C), P (45.4 MPa), OR T (60 °C), P (50.0 MPa),		71.6% β-CAR max. Rec 90.3% β-CRY max. Rec 36.4% ZEA max. Rec						[122]
	LY and homogenized	T (40–60 °C), P (20–40 MPa), CO_2_ Flow (0.8 g/min), t (180 min), Co-solv (EtOH 0–5% mol)	T (40 °C), P (40 MPa), CO_2_ Flow (0.8 g/min), t (180 min), Co-solv (EtOH 5% mol)		20.35 mg/g_extr_ β-CAR 25.96 mg/g_extr_ ZEA					193.75 mg/g_extr_ PA, 5.3 mg/g_extr_ PLA 71.96 mg/g_extr_ STA, 4.13 mg/g_extr_ OA, 94.66 mg/g_extr_ LNA, 2.95 mg/g_extr_ GLA	[17]
*T. chui*	Dried and GR	T (40–60 °C), P (18–25 MPa), CO_2_ Flow (2 mL/min), t (60–90 min), Co-solv (EtOH, MeOH)	T (40 °C), P (18 MPa), CO_2_ Flow (2 mL/min), t (60–90 min), Co-solv (MeOH)	4.3%							[124]
*Tetraselmis* sp.	LY and/or MW	T (45 °C), P (30 MPa), CO_2_ Flow (0.4 kg/h), t (90 min), Co-solv (EtOH 5%)		14.8%					11.1% Lipid Y		[93]
	LY	T (40 °C), P (15 MPa), CO_2_ Flow (5 mL/min), t (30 min), Co-solv (EtOH 5%)							10.88% Lipid Y		[125]

**Table 3:** Experimental design and kinetic modeling of SFE and other extraction methods compared to SFE.

Algae	Parametric Investigation	Ext. Yield/ Recovery	Kinetic Model	Experimental Design	Other Extraction Methods	Results	Ref.
*A. maxima*	T (50–60 °C), P (25–35 MPa), Co-solv (EtOH 0–10% *v*/*v*)				B-D	Total Lipids Determination	[24]
					Hexane MAC ( T = 25 °C, t = 2 h, stirring = 100 rpm)	2.6% wt lipid/biomass 0.01% wt GLA/biomass	
					EtOH MAC ( T = 25 °C, t = 2 h, stirring = 100 rpm)	5.7% wt lipid/biomass 0.68% wt GLA/biomass	
					ACE MAC ( T = 25 °C, t = 2 h, stirring = 100 rpm)	4.7% wt lipid/biomass 0.63% wt GLA/biomass	
	T (50–60 °C), P (25–35 MPa), CO_2_ Flow (2 g/min), t (390 min), Co-solv (EtOH 0–10% *v*/***v***)		internal mass transfer		Lepage and Roy	1.23% wt GLA/biomass	[25,26]
					B-D	7.8% wt lipid/biomass 0.98% wt GLA/biomass	
					Hexane MAC ( T = 25 °C, t = 2 h, stirring = 100 rpm)	2.6% wt lipid/biomass 0.01% wt GLA/biomass	
					EtOH MAC ( T = 25 °C, t = 2 h, stirring = 100 rpm)	5.7% wt lipid/biomass 0.68% wt GLA/biomass	
					ACE MAC ( T = 25 °C, t = 2 h, stirring = 100 rpm)	4.7% wt lipid/biomass 0.63% wt GLA/biomass	
	T (20–70 °C), P (15–18 MPa), CO_2_ Flow (3.33 × 10–^5^ kg/s), t (660 min)		Goto et al.-LDF	Two-level factorial design			[27]
*A. pacifica*	T (40–80 °C), P (15–35 MPa), CO_2_ Flow (2 mL/min), t (40–100 min), Co-solv (EtOH 5–15% *v*/*v*)			Two-level factorial design	Tetrahydrofuran/MeOH MAC	50 mg/100 g ZEA, 8 mg/100 g β-CRY, 120 mg/100 g β-CAR	[29]
*A. platensis*	T (45–60 °C), P (15–45 MPa), CO_2_Flow (0.015 kg/h), t (50 min), Co-solv (EtOH 26.70–53.22% *v*/*v*)	4.07%		Two-level factorial design	MAE with MeOH/EtA/light petroleum (1:1:1 *v/v/v*) (T = 50 °C, W = 40 W)	2.03% Y, 2.46 μg/g TOCs, 629 μg/g T.CAR, 15.88 mg/g FAs	[30]
	T (32–48 °C), P (20–40 MPa), t (120–240 min), Co-solv (EtOH)	10.26 g/kg		RSM, Box-Behnken design			[35]
	T (33.18–66.82 °C), P (23.2–56.8 MPa), CO_2_ Flow (0.24–0.9 kg/h), t (0–120 min soaking and 30–180 min Extr.) Co-solv (MeOH, ACE, EtA 0–10 mL, Aq.EtOH (20–80%) 5–28.4 mL)			RSM, CCD			[37]
	T (40–80 °C), P (10–30 MPa), t (30–90 min), Co-solv (EtOH 10–50% *v*/*v*)	6.7% *w*/*w*		Taguchi’s orthogonal array	PLE (T = 60–180 °C, P = 3.4–20.7 MPa, t = 5–15 min, ethyl lactate 0–100% *v*/*v*)	20.7% Y, 68.3% GLA Rec (in optimal conditions)	[31]
	T (40 °C), P (31.6–48.4 MPa), CO_2_ Flow (0.7 L/min), t (26.4–94 min), Co-solv (EtOH 9.64–16.36 mL)			RSM, CCD	B-D (UAE)	for GLA Rec	[32]
					MeOH/acetyl chloride MAC (T = 80 °C, 1 h)	for GLA Rec	
	T (60 °C), P (40 MPa), CO_2_ Flow (0.35 kg/h)	10.98%	Sovová				[38]
	T (40–55 °C), P (25–70 MPa), CO_2_ Flow (10 kg/h), t (90–240 min)	7.79% Lipid	Andrich et al.		Hexane MAC (t = 8 h)	7.77% Lipid Y	[39]
*B. braunii*	T (40 °C), P (12.5–30 MPa)				Hexane MAC	~76 g/kg Hydrocarbons	[24,40]
	T (50–80 °C), P (20–25 MPa), t (10–150 min)	~10.5%			B-D	18.2% FA Y	[41]
*C. protothecoides*	T (50 °C), P (35 MPa), CO_2_ Flow (0.0439 kg/h), t (180 min)	0.23 g/g_biom_ lipid 75% Rec	Goto et al.		SX (n-Hexane, t = 24 h)	0.32 g/g Lipid Y	[43]
	T (60 °C), P (30 MPa), CO_2_ Flow (30 g/h), t (90 min), Co-solv (EtOH 5%)	10% Lipid	Sovová & Semiemperical solubility models				[44]
*C. pyrenoidosa*	T (32–55 °C), P (25–40 MPa), CO_2_ Flow (15–30 kg/h), t (1.5–180 min), Co-solv (EtOH 0–1.5 mL/g_biom_)	7.78%		Orthogonal design (L_16_^45^)			[46]
*C. saccharophila*	T (42–73 °C), P (24.1–41.4 MPa), t (30–90 min)			RSM, Box-Behnken design			[48]
*C. sorokiniana*	T (40–60 °C), P (10–30 MPa), t (180 min), Co-solv (EtOH 0–10%)	35.03 mg/g		RSM, CCD	EtA and MeOH MAC	0.215 mg/g VIO Y 2.797 mg/g LUT Y 0.756 mg/g Carotene Y	[49]
*Chlorella* sp.	T (40–60 °C) P (15–30 MPa), CO_2_ Flow (15 g/min), t (180 min), Co-solv (Hexane/MeOH 1–3 *v*/*v*)	47.2%		RSM, Box-Behnken design			[51]
	T (60 °C), P (20–30 MPa), CO_2_ Flow (30 g/h), t (180 min), Co-solv (EtOH 0–5%)				B-D	15.2% Y	[52]
*C. vulgaris*	T (60–80 °C), P (20–50 MPa), CO_2_ Flow (2.5 mL/min), t (3–6 h), Co-solv (EtOH or ACE 7.5% *v/v*)				SX (EtOH, t = 5 h)	2 mg/g Extr LUT Y, 18 mg/g Extr CHL Y	[59]
	T (40–55 °C), P (15–35 MPa), CO_2_ Flow (0.4 dm^3^/min), t (125–480)				B-D	24.5% Lipid Y	[61]
					n-hexane MAC (t = 72 h)	0.03% Y	
					ACE MAC (t = 72 h)	0.04% Y	
	T (40 °C), P (12.5–30 MPa), CO_2_ Flow (0.04 kg/h)				ACE MAC	0.43% T.CAR Y	[40]
	T (50 °C), P (31 MPa), CO_2_ Flow (6 NL/min), t (20 min), Co-solv (Aq. EtOH (50%) 50 mL)	8.71%			UAE (0.5 g algae with 60 mL 50% aqueous EtOH, t = 15 h)	9.73% Y, 0.46 mg GAE/g _Extr_, 0.86 mg quercetin/g _Extr_	[65]
	T (40–60 °C), P (27.6–48.3 MPa), CO_2_ Flow (1–3 g/min), t (1–180 min)	17.7%	BICM, LDF, shrinking core model, BICM + shrinking core model	RSM, CCD	SX (n-hexane, t = 14 h)	18% Y	[55]
	T (40–80 °C), P (27.6–62.1 MPa), t (180 min)	19% > 99% Rec			SX (n-hexane, t = 12 h)	18% Y	[56]
	T (40–70 °C), P (20–28 MPa), CO_2_ Flow (10 kg/h), t (9 h)	4.86%		RSM, CCD			[57]
	T (40 °C), P (30 MPa), CO_2_ Flow (0.34–0.6 L/min) Co-solv (EtOH or oil)				Soybean oil MAC (T= ambient, t = 17 h or T= 100 °C, t = 30 min)	0.438% or 0.306% Y, 100% or 70.9% Rec	[58]
					ACE MAC	0.426% Y 100% Rec	
	T (45 °C), P (45 MPa), CO_2_ Flow (25 g/min)	~14 %	Sovová				[38]
	T (50 °C), P (25 MPa), CO_2_ Flow (0.5 kg/h), t (210–230 min) Co-solv (EtOH 0–10% *v/v*)	~40%			SX (CHF/MeOH 35:65 *v/v*, t = 18 h)	0.244 g/g Total Lipid Y 26% Neutral Lipid Rec 59% Glycolipid Rec 15% Phospholipid Rec	[63]
*Chlorococcum* sp.	T (60–80 °C), P (30 MPa), CO_2_ Flow (400 mL/min), t (80 min)	7.1% Lipid	Ozkal et al.		Hexane MAC (t = 7.5 h, T = ambient)	1.5% Lipid Y	[68]
					Hexane and hexane/isopropanol (3:2) MAC (t = 7.5 h, T = ambient)	1.0% Lipid Y	
					SX (Hexane, t = 7.5 h)	3.2% Lipid Y	
Commercial DHA algae	T (30–60 °C), P (10.5–30 MPa), CO_2_ Flow (20 mL/min), t (90–2700 min), Co-solv (EtOH, EtA, 1-Propanol 30:1–10:1)	90.56%			UAE (0.9 g algae, 48 mL EtA + 24 mL MeOH, T = 80 °C, t = 3 h)	for total lipid determination	[70]
*Crypthecodinium cohnii*	T (40–50 °C), P (20–30 MPa), CO_2_ Flow (0.6 kg/h), t (180 min)	8.6% Lipid			B-D	19.9% Lipid Y	[71]
*Cylindrotheca closterium*	T (60 °C), P (40 MPa), CO_2_ Flow (0.41 kg/h)	12.73%	Sovová				[38]
*D. salina*	T (40–60 °C), P (10–50 MPa), CO_2_ Flow(4.5 mmol/min), t (180 min), Co-solv (EtOH 0–5% mol)	1.2%	Reverchon et al.				[75]
	T (9.8–45.2 °C), P (18.5–44.2 MPa), t (100 min)			CCRD			[73]
	T (9.8–45.2 °C), P (18.5–44.2 MPa), t (100 min)	6.58%		CCRD			[72]
	T (40–60 °C), P (10–50 MPa), CO_2_ Flow (4.5 mmol/min) t (180 min)			Multi-level factorial design	UAE (0.105 g algae in 5 mL DMF, t = 3 min)	27.7 μg T.CAR/mg, 3.1 μg CHL/mg	[74]
					UAE (0.105 g algae in 5 mL MeOH, t = 3 min)	14.1 μg T.CAR/mg, 2.5 μg CHL/mg	
	T (40–60 °C), P (10–50 MPa), CO_2_ Flow (4.5 mmol/min) t (180 min), Co-solv (EtOH 5% mol)			Multi-level factorial design	UAE (t = 3 min, 5 mL MeOH, 0.025 g biomass)	14.1 μg/mg T.CAR, 2.5 μg/mg Total CHL	[76]
					UAE (t = 3 min, 5 mL DMF, 0.025 g biomass)	27.7 μg/mg T.CAR, 3.1 μg/mg Total CHL	
	T (30–60 °C), P (10–50 MPa), CO_2_ Flow (3 L/min), t (90 min)			RSM	MeOH MAC (t = 8 h, 2 g biomass, 150 mL)	245.74 μg/g T.CAR, 917.96 μg/g Total CHL	[77]
*H. pluvialis*	T (40–80 °C), P (30–50 MPa), t (60–240 min)			RSM, CCD			[92]
	T (40–80 °C), P (30–50 MPa), CO_2_ Flow (3 mL/min), t (60–240 min)			RSM, CCD	SX (ACE 250 mL, 0.5 g biomass, t = 6 h)	for total AST determination (27.46 mg/g)	[81]
	T (40–70 °C), P (30–55 MPa), t (300 min), Co-solv (EtOH 0–8% *v/v*)		Sovová	Two-level factorial design			[82]
	T (30–80 °C), P (6.9–34.5 MPa), CO_2_ Flow (2–12 ΝL/min) t (20–100 min), Co-solv (EtOH/Water 19.5–78 mL 0–99.5% *v/v*)			Design with 7 factors	SX (DCM 200 mL, 1.0 g biomass, T = 45 °C)	for total AST determination	[83]
	T (40–70 °C), P (35–75 MPa), CO_2_ Flow (10 g/min) t (270–600 min)				ACE MAC (multiple circles)	for total AST determination	[84]
	T (45 °C), P (11.7–48.3 MPa), CO_2_ Flow (2.7 mL/min) t (240 min)				B-D	for total TAG (366.3 mg for GR and 468.3 mg for homogenized biomass)	[85]
					ACE MAC	for total AST (41.4 mg for GR and 71.0 mg for homogenized biomass)	
	T (40–60 °C), P (20–30 MPa), Co-solv (EtOH 0–10%)				ACE MAC	for T.CAR determination (1.80% Y, 3.3% LUT, 2.2% CAN, 7.2% β-CAR, 75.0% Total AST)	[86]
	T (40–80 °C), P (20–55 MPa), CO_2_ Flow (2–4 mL/min), t (240 min), Co-solv(EtOH 0–7.5% *v/v*)				SX (DCM 200 mL, 6 g biomass, t = 6 h)	for total AST Rec (3.43% AST Y)	[87]
	T (50–80 °C), P (30–50 MPa), CO_2_ Flow (2–4 mL/min), t (300 min), Co-solv (EtOH/Soy bean oil/Olive oil 0–12% *v/v*)				SX (DCM 200 mL, 1 g biomass, t = 2 h)	for total AST Rec	[88]
	T (40–70 °C), P (20–35 MPa), CO_2_ Flow (0.06 g/min), t (120 min), Co-solv (EtOH 0–13% *w/w*)	282.5 mg/g		RSM, Box-Behnken design	CO_2_ - Expanded EtOH (30–60 °C, EtOH 50–70% *w/w*, 7 MPa)	333.1 mg/g Y, 62.57 mg/g AST Content, 124.2% *w*/*w* AST Rec, 0.233 mM TE/g	[90]
*I. galbana*	T (40–60 °C), P (10–30 MPa), CO_2_ Flow (5 L/min), t (60 min)	5%		Factorial design	Reyes (ACE/BHT (99.9:0.01) 20 mL, t = 24 h, 200 mg biomass)	for total extr. compounds determination	[94]
					GXL ( T = 50 °C, P = 7 MPa, EtOH 15–75%)	as stage 2 - for enhanced CAR and CHL extr.	
					EtOH MAC ( T = 80 °C, P = 10 MPa)	as stage 3 - for mid- and highly-polar lipids, proteins and sugars extr.	
					Water MAC ( T = 80 °C, P = 10 MPa)	as stage 4 - for protein and sugars extr.	
*Isochrysis* sp.	T (45 °C), P (30 MPa), CO_2_ Flow (0.4 kg/h), t (120 min) Co-solv (EtOH 5%)	15.5%	Sovová		SX (MeOH/CHF (2:1), t = 18 h, T = 105 °C)	23.1% Y, 31.2% Free FA Conversion, 7.2% Lipid Y	[93]
					Kochert (MeOH/CHF (2:1), t = 1 h, T = 45 °C)	12.7% Y	
*Monoraphidium* sp.	T (30–60 °C), P (20 MPa), t (15–60 min), Co-solv (EtOH 0–20 mL)				Bead beater method (BBM) (ACE/hexane (35:65) 500 μL, 30 mg biomass)	2.44 mg/g AST, 100% AST Rec, 27.6 mg/g Total CHL, 100% Total CHL Rec	[95]
					EtOH MAC (20 mL, 1 g biomass, t = 30 min)	1.16 mg/g AST, 48% AST Rec, 16.1 mg/g Total CHL, 56% Total CHL Rec	
*N. gaditana*	T (40–60 °C), P (10–50 MPa), CO_2_ Flow (4.5 mmol/min) t (180 min)			Multilevel factorial Design	UAE MeOH (5 mL, 0.2 g biomass, t = 10 min, T = 4 °C, t = 24 h)	0.8 μg/mg T.CAR Y 18.5 μg/mg CHL-a Y	[96]
	T (40–60 °C), P (20–50 MPa), CO_2_ Flow (4.5 mmol/min)t (180 min) Co-solv (EtOH 5% mol)			Multilevel factorial design	UAE MeOH (5 mL, 0.2 g biomass, t = 10 min, T = 4 °C, t = 24 h)	2.2 μg/mg T.CAR Y, 26.4 μg/mg T.CHL Y	[76]
					UAE DMF (5 mL, 0.2 g biomass, t = 10 min, T = 4 °C, t = 24 h)	6.9 μg/mg T.CAR Y 41.5 μg/mg T.CHL Y	
	T (45 °C), P (30 MPa), CO_2_ Flow (0.4 kg/h), t (120 min) Co-solv (EtOH 5%)	12.9%	Sovová		SX (MeOH/CHF (2:1), t = 18 h, T = 105 °C)	23.1% Y, 31.2% Free FA Conversion, 7.2% Lipid Y	[93]
					Kochert (MeOH/CHF (2:1), t = 1 h, T = 45 °C)	12.7% Y	
	T (40–60 °C), P (20–50 MPa), CO_2_ Flow (4.5 mmol/min) t (180 min) Co-solv (EtOH 0–5% mol)		Reverchon et al.				[75]
	T (55 °C), P (40 MPa), CO_2_ Flow (10 L/min), t (270 min)	11.48%	Sovová		PLE (water or EtOH/water (1:1) or EtOH, T = 40–170 °C, t = 20 min)	37.71% Y, 9.04 mg/g Extr T.CAR Y, 69.14% Lipid Y, 59.85 mg GAE/g Extr, 0.8 mmol TE/g Extr (optimum conditions)	[97]
					ACE MAC (t = 24 h)	for total VIO determination	
*N. granulata*	T (50–90 °C), P (35–55 MPa), CO_2_ Flow (100 g/min), t (180–270 min)	28.45 mg/g ash free biomass			SX (hexane, 0.5 g biomass, t = 1 h)	57.34 mg/g Y, 17.35 mg/g FAME	[99]
*N. oculata*	T (50 °C), P (25–35 MPa), CO_2_ Flow (20 mL/min), Co-solv (EtOH, DCM, Toluene, n-Hexane)				SX (hexane 300 mL, 10 g biomass, t = 16 h)	5.79% Y, 56.3% CAR Rec	[101]
					SX (EtOH 300 mL, 10 g biomass, t = 16 h)	40.90% Y, 70.3% CAR Rec	
					SX (DCM 300 mL, 10 g biomass, t = 16 h)	9% Y, 100% CAR Rec	
	T (40–80 °C), P (20.7–62.1 MPa), CO_2_ Flow (24 mL/min), t (240 min)	47.30 mg/g			Chen method (hexane 1 mL, 5 mg biomass)	for TOC Rec 4.722 mg/g_extr_, 163 mg/g Y	[103]
					Cequier-Sanchez method (DCM/MeOH)	665.33 mg/g Y, Composition 74.63 mg/g Total SFA 23.41 mg/g Total MUFA 1.96 mg/g Total PUFA	
	T (60 °C), P (30–85 MPa), CO_2_ Flow (0.5–100 kg/h), t (270 min)	~15%			B-D	Composition 0.71% Free FA 72.13% Triglycerides 4.58% Sterol	[102]
	T (60 °C), P (40 MPa), CO_2_ Flow (0.4–0.5 kg/h), t (120 min)	~12%	Sovová				[38]
*N. salina*	T (60 °C), P (30 MPa), CO_2_ Flow (0.4 kg/h), t (90 min), Co-solv (EtOH 5%)	~30%	Sovová				[44]
*Nannochloropsis* sp.	T (40–55 °C), P (40–70 MPa), CO_2_ Flow (10 kg/h), t (360 min)	~257 mg/g Lipid			SX (hexane, t = 6 h)	237 mg/g Lipid Y, 25.6% SFA Comp., 21.9% Monoenoic Comp., 52.2% PUFA Comp., 42.6% n-3 PUFAs Comp.	[104]
	T (50–75 °C), P (10–55 MPa), CO_2_ Flow(7.2–14.5 g/min) t (100 min)	94.28 mg/g OR 58.26 mg/g			B-D	for total lipid determination	[106]
	T (40–60 °C), P (12.5–30 MPa), CO_2_ Flow(0.35–0.62 g/min) t (60–105 min), Co-solv (EtOH 0–20% *w*/*w*)				B-D method (MeOH/CHF/H_2_ O (10:5:4 *v/v/v*), 150 mg biom., t = 24 h)	25.3% Lipid Y	[105]
					SX (hexane, 1 g biom., t = 6 h)	40.7% Lipid Y	
					SX (EtOH, 1 g biom., t = 6 h)	50.6% Lipid Y	
					EtA MAC (19 mL, 1 g biom., t = 24 min, T = 65 °C)	for T.CAR determination	
					EtA or ACE MAC (2 mL, 5 g biom., t = 10 min, T =−22 °C)	for T.CAR determination	
*Pavlova* sp.	T (45 °C), P (30.6 MPa), t (360 min)	17.9%			UAE (10 mL water/24 mL MeOH/48 mL EtA, 10 g biom., t = 3 h)	44.7% Y, 15.6% (98.1% Rec) FAME	[108]
					SX (hexane 450 mL, 2 g biom., t = 15 h)	13.5% Y, 7.2% (45.2% Rec) FAME	
					SX (hexane 450 mL, 2 g biomass, t = 100 h)	18.5% Y, 9.8% (61.6% Rec) FAME	
					SX (hexane 450 mL, 2 g biom., t = 15 h, bead milled)	15.3% Y, 9.3% (58.5% Rec) FAME	
*Phaeodactylum* *tricornutum*	T (45 °C), P (30.6 MPa), CO_2_ Flow (2.5 L/min), t (360 min)				B-D method (3 mL MeOH/CHF 1:2 *v/v*, 100 mg biom., t = 2 h, T = 50 °C)	31.3% Lipid Y, 11.1% TFA Y, 2.0% EPA Y, 4.4% PUFA Y	[109]
					DMC MAC (3 mL, 100 mg biom., t = 2 h, T = 50 °C)	11.3% Lipid Y, 4.5% TFA Y, 1.1% EPA Y, 2.6% PUFA Y	
					DMC MAC (3 mL, 100 mg biom., t = 2 h, T = 50 °C, DES pretreated)	14.1% Lipid Y, 8.1% TFA Y, 1.6% EPA Y, 3.6% PUFA Y	
					DMC MAC (3 mL, 100 mg biom., t = 2 h, T = 50 °C, MW and DES pretreated for t = 30 min, T = 150 °C)	9.2% Lipid Y, 3.9% TFA Y, 2.2% EPA Y, 4.4% PUFA Y	
					DMC MAC (3 mL, 100 mg biom., t = 2 h, T = 50 °C, MW and DES pretreated for t = 60 min, T = 100 °C)	12.5% Lipid Y, 11.0% TFA Y, 2.2% EPA Y, 4.6% PUFA Y	
*Phormidium* *valderianum*	T (35.86–64.14 °C), P (13.79–56.21 MPa), CO_2_ Flow (2 L/min), t (90 min)	3.96 mg/g		CCRD	SX (hexane, 10 g biomass, t = 8 h)	125.15 μg GAE/g TPC, 19.21 μg β-CAR eq./g T.CAR, 2451 μg BH equivalent/g Reducing power	[110]
*S. almeriansis*	T (32–60 °C), P (20–60 MPa), CO_2_ Flow (1 g/min), t (300 min)			RSM	ACE MAC	2.33 mg/g LUT, 3.07 mg/g β-CAR	[111]
	T (50–65 °C), P (25–55 MPa), CO_2_ Flow(7.2–14.5 g/min)t (120 min)	8.74 mg/g			B-D (3.75 mL MeOH/CHF 2:1, 120 mg biomass, t = 1 h)	for lipid determination	[112]
	T (45 °C), P (30 MPa), CO_2_ Flow (0.4 kg/h), t (90 min), Co-solv (EtOH 5% *v/v*)	13.2%	Sovová		Kochert Method (MeOH/CHF 1:2 *v*/*v*, t = 1 h, T = 45 °C)	15.7% Y	[93]
					SX (MeOH/CHF 2:1 *v*/*v*, t = 18 h)	22.4% Y, 8.0% Lipid Y, 35.7% Free FA Conversion	
*S. dimorphus*	T (50–100 °C), P (16.6–50 MPa), t (60 min)				B-D	for total lipid determination	[113]
*S. obliquus*	T (40–60 °C), P (10–40 MPa), CO_2_ Flow (7 L/min), t (120 min)	0.97%		RSM	Axelsson-Gentili method (MeOH/CHF 1:2 *v*/*v* 8 mL, 25 mg biomass)	for total lipid determination	[114]
					ACE MAC (20 mL with BHT 0.1% *w*/*v*, t = 24 h, 200 mg biomass)	for total extr. compounds determination	
					PLE (EtOH 0–100%, T = 50–170 °C, P = 70 MPa)	4.83–78.04% Y, 0.66–124.1 mg/g_extr_ CHL, 6.3–49.41 mg GAE/g_extr_ TPC 0.11–1.6 mmol TE/ g_extr_ AO	
	T (45–65 °C), P (15–30 MPa), CO_2_ Flow (0.4 kg/h), t (30–90 min), Co-solv (EtOH 5% *v*/*v* )	24.67%	Sovová		SX (MeOH/CHF 2:1 *v*/*v*, t = 18 h)	29.03% Y, 51.13% Free FA Conv., 14.84% Lipid Y, 27.38% SFA, 19.95% MUFA, 52.67% PUFA, 36.32% Ω-3, 11.20% Ω-6	[44]
	T (20–200 °C), P (7–80 MPa), t (540 min)	6.4%			B-D (with hexane, t = 8 h)	for total lipid determination	[2]
	T (40–60 °C), P (15–25 MPa), CO_2_ Flow (2–4.3 g/min), t (240 min), Co-solv (EtOH 0–9.5% *v*/*v* )				ACE MAC (5 mL, t = 2 h)	24.00 mg/g CHL-a, 19.04 mg/g CHL-b, 18.90 mg/g CHL-c, 17.78 mg/g T.CAR	[116]
*S. obtusiusculus*	T (20 °C), P (12 MPa), t (540 min)	6.4%			B-D (with hexane, t = 8 h)	for total lipid determination	[2]
*Scenedesmus* sp.	T (35–80 °C), P (20–40 MPa), CO_2_ Flow (750–800 mL/min), t (60 min), Co-solv (MeOH, EtOH, Propanol, Butanol, ACE 0–40% mol)				MeOH MAC	0.388 mg/g LUT Y	[118]
					EtOH MAC	0.345 mg/g LUT Y	
					Propanol MAC	0.291 mg/g LUT Y	
					Butanol MAC	0.269 mg/g LUT Y	
					ACE MAC	0.3579 mg/g LUT Y	
	T (60 °C), P (30 MPa), CO_2_ Flow (2 mL/min), t (60 min), Co-solv (EtOH 0–10% mol)				UAE (hexane 40 mL,1 g biom.)	4.00% lipid	[119]
					UAE (CHF/MeOH/H_2_O 1:1:0.9 *v*/*v* 40 mL, 1 g biomass)	4.26% Lipid Y	
					UAE (n-hexane/iso-propanol 3:2 *v*/*v* 40 mL, 1 g biomass)	4.62% Lipid Y	
	T (35–65 °C), P (20–50 MPa), CO_2_ Flow (1.38–4.02 g/min)	7.06%		Multilevel Factorial Design	B-D (hexane 40 mL, 1 g biomass, UAE, t = 30 min)	4.00% Lipid Y	[120]
					B-D (CHF/MeOH/Water 1:1:0.9 *v*/*v*/*v* 40 mL, 1 g biomass, UAE, t = 30 min)	4.26% Lipid Y	
					B-D (hexane/isopropanol 3:2 *v*/*v* 40 mL, 1 g biomass, UAE, t = 30 min)	4.62% Lipid Y	
					Folch (Hexane, CHF/MeOH/Water 1:1:0.9 *v*/*v*/*v*, Hexane/Isopropanol 3:2 *v*/*v*, 40 mL, 1 g biomass, UAE, t = 30 min)	for total lipid determination	
					Hara & Radin (Hexane, CHF/MeOH/Water 1:1:0.9 *v*/*v*/*v*, Hexane/Isopropanol 3:2 *v*/*v*, 40 mL, 1 g biomass, UAE, t = 30 min)	for total lipid determination	
					SX (Hexane 75 mL, 1 g biomass, t = 12 h)	2.61% Lipid Y	
	T (35–50 °C), P (40 MPa), t (120–360 min), Co-solv (MeOH)				Folch (MeOH/CHF 1:2 *v*/*v* )	5.8% Lipid Y	[121]
*Synechococcus* sp.	T (40–60 °C), P (20–50 MPa), CO_2_ Flow (4.5 mmol/min), t (180 min), Co-solv (EtOH 0–5% mol)		Reverchon et al.				[75]
	T (40–60 °C), P (20–50 MPa), CO_2_ Flow (4.5 mmol/min), t (240 min), Co-solv (EtOH 15% mol)			Multilevel Factorial Design	UAE DMF (1 mL, 2–5 mg)	5.4 mg/g CHL, 0.48 mg/g MYX, 2.15 mg/g β-CAR, 0.12 mg/g β-CRY, 1.79 mg/g ZEA, 4.93 mg/g T.CAR	[122]
	T (40–60 °C), P (20–50 MPa), CO_2_ Flow (4.5 mmol/min), t (180 min), Co-solv (EtOH 5% mol)				UAE DMF (5 mL, 0.1 g)	3.3 μg/mg Total T.CAR, 9.6 μg/mg Total CHL	[76]
					UAE MeOH (5 mL, 0.1 g)	1.4 μg/mg T.CAR, 4.1 μg/mg Total CHL	
	T (40–60 °C), P (20–50 MPa), CO_2_ Flow (4.5 mmol/min), t (180 min)			Multilevel Factorial Design	UAE MeOH (5 mL, 0.1 g, t = 10 min)	1.353 μg/mg T.CAR, 4.096 μg/mg Total CHL	[123]
	T (40–60 °C), P (20–40 MPa), CO_2_ Flow (0.8 g/min), t (180 min), Co-solv (EtOH 0–5% mol)				UAE (DMF 5 mL, 0.105 g biomass, t = 3 min)	42.53 mg/g_extr_ β-CAR, 10.09 mg/g_extr_ ZEA 59.38 mg/g_extr_ PA, 8.89 mg/g_extr_ PLA, 6.47 mg/g_extr_ OA, 2.11 mg/g_extr_ LOA, 0.23 mg/g Extr LNA	[17]
*T. chui*	T (40–60 °C), P (18–25 MPa), CO_2_ Flow (2 mL/min), t (60–90 min), Co-solv (EtOH, MeOH)	4.3%			ASE (DCM/MeOH 9:1, t = 60 min)	14.6% Y	[124]
*Tetraselmis* sp.	T (45 °C), P (30 MPa), CO_2_ Flow (0.4 kg/h), t (90 min), Co-solv (EtOH 5%)	14.8%		Sovová	SX (MeOH/CHF 2:1, t = 18 h, T = 105 °C)	17.7% Y, 38.7% Free FA Conv., 7.0% Lipid Y	[93]
					Kochert (MeOH/CHF 2:1, t = 1 h, T = 45 °C)	19.1% Y	
	T (40 °C), P (15 MPa), CO_2_ Flow (5 mL/min), t (30 min), Co-solv (EtOH 5%)				B-D (MeOH/CHF 2:1 *v*/*v* 5 mL, 200 mg biomass, t = 4 h)	11.66% Lipid Y	[125]
					Cequier-Sanchez (MeOH/DCM 1:2 *v*/*v* 6–8 mL, 200 mg biomass, t = 2 h)	15.05% Lipid Y	
					Schlechtriem (Propan-2-ol/Cyclohexane 1:1.25 *v*/*v* 9 mL, 200 mg biomass, UAE, t = 30 min)	13.35% Lipid Y	
					Burja (3 mM KOH in 96% EtOH 15.2 mL, 200 mg biomass, UAE, t = 1 h)	9.40% Lipid Y	

## Data Availability

Not applicable.

## Abbreviations

AA	Arachidic acid
ACE	Acetone
AM	Antimicrobial activity
AO	Antioxidant activity (IC_50_)
ASE	Accelerated Solvent Extractor
AST	Astaxanthin
B-D	Bligh and Dyer
BICM	Broken Plus Intact Cell Model
CAN	Canthaxanthin
CCD	Central Composite Design
CCRD	Central Composite Rotatable Design
CHF	Chloroform
CHL-a	Chlorophyll a
CHL-b	Chlorophyll b
CHL-c	Chlorophyll c
Comp.	Composition
Co-Solv	Co-solvent
CRY	Cryptoxanthin
DCM	Dichloromethane
DHA	Docosahexaenoic Acid
DMF	Dimethylformamide
DMC	Dimethyl Carbonate
DPPH	2,2-diphenyl-1-picrylhydrazyl
EPA	Eicosapentaenoic Acid
EtA	Ethyl Acetate
EtOH	Ethanol
Extr	Extract
FA	Fatty Acids
FAME	Fatty Acid Methyl Esters
GAE	Gallic Acid Equivalent
GXL	Gas Expanded Liquid
GLA	γ-Linolenic Acid
GR	Ground
IC_50_/EC_50_	50% Inhibition
LA	Linolic Acid
LAA	Lauric acid
LDF	Linear Driving Force Model
LNA	Linolenic Acid
LUT	Lutein
LY	Lyophilized
MA	Myristic acid
MAC	Maceration
MAE	Microwave Assisted Extraction
MBC	Minimum Bactericidal Concentration
MeOH	Methanol
MFC	Minimal Fungicidal Concentration
MUFA	Monounsaturated Fatty Acids
MW	Microwave
MYX	Myxoxanthophyll
NEO	Neoxanthin
OA	Oleic Acid
P	Pressure
PA	Palmitic Acid
PHY	Phycocyanine
PLA	Palmitoleic Acid
PLE	Pressurized Liquid Extraction
PUFA	Polyunsaturated Fatty Acids
Rec	Recovery
RSM	Response Surface Methodology
SEP	Separator
SFA	Saturated Fatty Acids
SFE	Supercritical Fluid Extraction
STA	Stearic Acid
STP	Standard Temperature and Pressure
SX	Soxhlet
t	Time/Duration
T	Temperature
T.CAR	Total Carotenoids
T.CHL	Total Chlorophyll
TE	Trolox Equivalent
TFA	Total Fatty Acids
TOC	Tocopherol
TPC	Total Phenolic Content
UAE	Ultrasound Assisted Extraction
VAU	Vaucheriaxanthin
VIO	Violaxanthin
Y	Yield
ZEA	Zeaxanthin
β-CAR	β-Carotene

## References

[1] Picot-Allain C., Mahomoodally M.F., Ak G., Zengin G. (2021). Conventional versus green extraction techniques—A comparative perspective. Curr. Opin. Food Sci..

[2] Lorenzen J., Igl N., Tippelt M., Stege A., Qoura F., Sohling U., Bruck T. (2017). Extraction of microalgae derived lipids with supercritical carbon dioxide in an industrial relevant pilot plant. Bioprocess. Biosyst. Eng..

[3] Silva S.C., Ferreira I.C.F.R., Dias M.M., Barreiro M.F. (2020). Microalgae-Derived Pigments: A 10-Year Bibliometric Review and Industry and Market Trend Analysis. Molecules.

[4] Daneshvar E., Sik Ok Y., Tavakoli S., Sarkar B., Shaheen S.M., Hong H., Luo Y., Rinklebe J., Song H., Bhatnagar A. (2021). Insights into upstream processing of microalgae: A review. Bioresour. Technol..

[5] Vale M.A., Ferreira A., Pires J.C.M., Gonçalves A.L., Rahimpour M.R., Farsi M., Makarem M.A. (2020). Chapter 17—CO_2_ capture using microalgae. Advances in Carbon Capture.

[6] Borowitzka M.A. (2013). High-value products from microalgae—Their development and commercialisation. J. Appl. Phycol..

[7] Borowiak D., Krzywonos M. (2022). Bioenergy, Biofuels, Lipids and Pigments—Research Trends in the Use of Microalgae Grown in Photobioreactors. Energies.

[8] Gong M., Bassi A. (2016). Carotenoids from microalgae: A review of recent developments. Biotechnol. Adv..

[9] Yen H.-W., Yang S.-C., Chen C.-H., Jesisca, Chang J.-S. (2015). Supercritical fluid extraction of valuable compounds from microalgal biomass. Bioresour. Technol..

[10] Chemat F., Vian M.A., Cravotto G. (2012). Green Extraction of Natural Products: Concept and Principles. Int. J. Mol. Sci..

[11] Mandal S.C., Mandal V., Das A.K., Mandal S.C., Mandal V., Das A.K. (2015). Chapter 6—Classification of Extraction Methods. Essentials of Botanical Extraction.

[12] Leonelli C., Veronesi P., Cravotto G., Chemat F., Cravotto G. (2013). Microwave-Assisted Extraction: An Introduction to Dielectric Heating. Microwave-assisted Extraction for Bioactive Compounds: Theory and Practice.

[13] Al-Nimer M., Wahbee Z. (2017). Ultraviolet light assisted extraction of flavonoids and allantoin from aqueous and alcoholic extracts of *Symphytum officinale*. J. Intercult. Ethnopharmacol..

[14] Hitchen S.M., Dean J.R. (1993). Properties of Supercritical Fluids. Applications of Supercritical Fluids in Industrial Analysis.

[15] Ghasemi E., Raofie F., Najafi N.M. (2011). Application of response surface methodology and central composite design for the optimisation of supercritical fluid extraction of essential oils from *Myrtus communis* L. leaves. Food Chem..

[16] Uwineza P.A., Waśkiewicz A. (2020). Recent Advances in Supercritical Fluid Extraction of Natural Bioactive Compounds from Natural Plant Materials. Molecules.

[17] Cardoso L.C., Serrano C.M., Rodríguez M.R., de la Ossa E.J.M., Lubián L.M. (2012). Extraction of Carotenoids and Fatty Acids from Microalgae using Supercritical Technology. Am. J. Anal. Chem..

[18] Cassanelli M., Prosapio V., Norton I., Mills T. (2018). Design of a Cost-Reduced Flexible Plant for Supercritical Fluid-Assisted Applications. Chem. Eng. Technol..

[19] Tabernero A., Martín del Valle E.M., Galan M.A. (2013). Microalgae Technology: A Patent Survey. Int. J. Chem. React. Eng..

[20] WIPO (2016). W.I.P.O. Patent Landscape Report on Microalgae-Related Technologies.

[21] Ambati R.R., Gogisetty D., Aswathanarayana R.G., Ravi S., Bikkina P.N., Bo L., Yuepeng S. (2019). Industrial potential of carotenoid pigments from microalgae: Current trends and future prospects. Crit. Rev. Food Sci. Nutr..

[22] Maltsev Y., Maltseva K. (2021). Fatty acids of microalgae: Diversity and applications. Rev. Environ. Sci. Bio/Technol..

[23] Lang I., Hodac L., Friedl T., Feussner I. (2011). Fatty acid profiles and their distribution patterns in microalgae: A comprehensive analysis of more than 2000 strains from the SAG culture collection. BMC Plant Biol..

[24] Mendes R.L., Nobre B.P., Cardoso M.T., Pereira A.P., Palavra A.F. (2003). Supercritical carbon dioxide extraction of compounds with pharmaceutical importance from microalgae. Inorg. Chim. Acta.

[25] Mendes R.L., Reis A.D., Pereira A.P., Cardoso M.T., Palavra A.F., Coelho J.P. (2005). Supercritical CO_2_ extraction of γ-linolenic acid (GLA) from the cyanobacterium *Arthrospira* (Spirulina) *maxima*: Experiments and modeling. Chem. Eng. J..

[26] Mendes R.L., Reis A.D., Palavra A.F. (2006). Supercritical CO_2_ extraction of γ-linolenic acid and other lipids from *Arthrospira* (Spirulina) *maxima*: Comparison with organic solvent extraction. Food Chem..

[27] Canela A.P.R.F., Rosa P.T.V., Marques M.O.M., Meireles M.A.A. (2002). Supercritical Fluid Extraction of Fatty Acids and Carotenoids from the Microalgae *Spirulina maxima*. Ind. Eng. Chem. Res..

[28] Valderrama J.O., Perrut M., Majewski W. (2003). Extraction of Astaxantine and Phycocyanine from Microalgae with Supercritical Carbon Dioxide. J. Chem. Eng. Data.

[29] Careri M., Furlattini L., Mangia A., Musci M., Anklam E., Theobald A., von Holst C. (2001). Supercritical fluid extraction for liquid chromatographic determination of carotenoids in *Spirulina Pacifica* algae: A chemometric approach. J. Chromatogr. A.

[30] Esquivel-Hernandez D.A., Lopez V.H., Rodriguez-Rodriguez J., Aleman-Nava G.S., Cuellar-Bermudez S.P., Rostro-Alanis M., Parra-Saldivar R. (2016). Supercritical Carbon Dioxide and Microwave-Assisted Extraction of Functional Lipophilic Compounds from *Arthrospira platensis*. Int. J. Mol. Sci..

[31] Golmakani M.-T., Mendiola J.A., Rezaei K., Ibáñez E. (2012). Expanded ethanol with CO_2_ and pressurized ethyl lactate to obtain fractions enriched in γ-Linolenic Acid from *Arthrospira platensis* (Spirulina). J. Supercrit. Fluids.

[32] Sajilata M.G., Singhal R.S., Kamat M.Y. (2008). Supercritical CO_2_ extraction of γ-linolenic acid (GLA) from *Spirulina platensis* ARM 740 using response surface methodology. J. Food Eng..

[33] Qiuhui H. (1999). Supercritical Carbon Dioxide Extraction of Spirulina platensis Component and Removing the Stench. J. Agric. Food Chem..

[34] Mendiola J.A., Marín F.R., Hernández S.F., Arredondo B.O., Señoráns F.J., Ibañez E., Reglero G. (2005). Characterization via liquid chromatography coupled to diode array detector and tandem mass spectrometry of supercritical fluid antioxidant extracts of *Spirulina platensis* microalga. J. Sep. Sci..

[35] Wang L., Pan B., Sheng J., Xu J., Hu Q. (2007). Antioxidant activity of *Spirulina platensis* extracts by supercritical carbon dioxide extraction. Food Chem..

[36] Mendiola J.A., Jaime L., Santoyo S., Reglero G., Cifuentes A., Ibañez E., Señoráns F.J. (2007). Screening of functional compounds in supercritical fluid extracts from *Spirulina platensis*. Food Chem..

[37] Tong Y., Gao L., Xiao G., Pan X. (2011). Supercritical CO_2_ Extraction of Chlorophyll a from *Spirulina platensis* with a Static Modifier. Chem. Eng. Technol..

[38] Mouahid A., Crampon C., Toudji S.-A.A., Badens E. (2013). Supercritical CO_2_ extraction of neutral lipids from microalgae: Experiments and modelling. J. Supercrit. Fluids.

[39] Andrich G., Zinnai A., Nesti U., Venturi F. (2006). Supercritical fluid extraction of oil from microalga *Spirulina* (arthrospira) *platensis*. Acta Aliment..

[40] Palavra A.M.F., Coelho J.P., Barroso J.G., Rauter A.P., Fareleira J.M.N.A., Mainar A., Urieta J.S., Nobre B.P., Gouveia L., Mendes R.L. (2011). Supercritical carbon dioxide extraction of bioactive compounds from microalgae and volatile oils from aromatic plants. J. Supercrit. Fluids.

[41] Santana A., Jesus S., Larrayoz M.A., Filho R.M. (2012). Supercritical Carbon Dioxide Extraction of Algal Lipids for the Biodiesel Production. Procedia Eng..

[42] Mendiola J.A., Torres C.F., Toré A., Martín-Álvarez P.J., Santoyo S., Arredondo B.O., Señoráns F.J., Cifuentes A., Ibáñez E. (2006). Use of supercritical CO_2_ to obtain extracts with antimicrobial activity from *Chaetoceros muelleri* microalga. A correlation with their lipidic content. Eur. Food Res. Technol..

[43] Chen Y.H., Walker T.H. (2012). Fed-batch fermentation and supercritical fluid extraction of heterotrophic microalgal *Chlorella protothecoides* lipids. Bioresour. Technol..

[44] Solana M., Rizza C.S., Bertucco A. (2014). Exploiting microalgae as a source of essential fatty acids by supercritical fluid extraction of lipids: Comparison between *Scenedesmus obliquus*, *Chlorella protothecoides* and *Nannochloropsis salina*. J. Supercrit. Fluids.

[45] Viguera M., Marti A., Masca F., Prieto C., Calvo L. (2016). The process parameters and solid conditions that affect the supercritical CO_2_ extraction of the lipids produced by microalgae. J. Supercrit. Fluids.

[46] Hu Q., Pan B., Xu J., Sheng J., Shi Y. (2007). Effects of supercritical carbon dioxide extraction conditions on yields and antioxidant activity of *Chlorella pyrenoidosa* extracts. J. Food Eng..

[47] Wu Z., Wu S., Shi X. (2007). Supercritical Fluid Extraction and Determination of Lutein in Heterotrophically Cultivated *Chlorella Pyrenoidosa*. J. Food Process Eng..

[48] Alhattab M., Kermanshahi-pour A., Su-Ling Brooks M. (2019). Dispersed air flotation of *Chlorella saccharophila* and subsequent extraction of lipids—Effect of supercritical CO_2_ extraction parameters and surfactant pretreatment. Biomass Bioenergy.

[49] Morcelli A., Cassel E., Vargas R., Rech R., Marcílio N. (2021). Supercritical fluid (CO_2_+ethanol) extraction of chlorophylls and carotenoids from *Chlorella sorokiniana*: COSMO-SAC assisted prediction of properties and experimental approach. J. CO2 Util..

[50] Abrahamsson V., Jumaah F., Turner C. (2018). Continuous multicomponent quantification during supercritical fluid extraction applied to microalgae using in-line UV/Vis absorption spectroscopy and on-line evaporative light scattering detection. J. Supercrit. Fluids.

[51] Char J.-M., Wang J.-K., Chow T.-J., Chien Q.-C. (2011). Biodiesel Production from Microalgae through Supercritical Carbon Dioxide Extraction. J. Jpn. Inst. Energy.

[52] Safi C., Camy S., Frances C., Varela M.M., Badia E.C., Pontalier P.-Y., Vaca-Garcia C. (2013). Extraction of lipids and pigments of *Chlorella vulgaris* by supercritical carbon dioxide: Influence of bead milling on extraction performance. J. Appl. Phycol..

[53] Zhou D., Qiao B., Li G., Xue S., Yin J. (2017). Continuous production of biodiesel from microalgae by extraction coupling with transesterification under supercritical conditions. Bioresour. Technol..

[54] Tai D.C., Hai D.T.T., Vinh N.H., Phung L.T.K. (2016). Extraction fatty acid as a source to produce biofuel in microalgae *Chlorella* sp. and *Spirulina* sp. using supercritical carbon dioxide. AIP Conf. Proc..

[55] Bahadar A., Khan M., Asim M., Jalwana K. (2015). Supercritical Fluid Extraction of Microalgae (*Chlorella vulagaris*) Biomass. Handbook of Marine Microalgae: Biotechnology Advances.

[56] Bahadar A., Khan M.B., Willmann J.C. (2016). Accelerated production and analysis of biofuel derived from photobioreactor engineered microalgae using super critical fluid extraction. Energy Sources Part A Recovery Util. Environ. Eff..

[57] Dejoye C., Vian M.A., Lumia G., Bouscarle C., Charton F., Chemat F. (2011). Combined extraction processes of lipid from *Chlorella vulgaris* microalgae: Microwave prior to supercritical carbon dioxide extraction. Int. J. Mol. Sci..

[58] Gouveia L., Nobre B.P., Marcelo F.M., Mrejen S., Cardoso M.T., Palavra A.F., Mendes R.L. (2007). Functional food oil coloured by pigments extracted from microalgae with supercritical CO_2_. Food Chem..

[59] Kitada K., Machmudah S., Sasaki M., Goto M., Nakashima Y., Kumamoto S., Hasegawa T. (2009). Supercritical CO_2_ extraction of pigment components with pharmaceutical importance from *Chlorella vulgaris*. J. Chem. Technol. Biotechnol..

[60] Moradi-kheibari N., Ahmadzadeh H. (2017). Supercritical carbon dioxide extraction and analysis of lipids from *Chlorella vulgaris* using gas chromatography. J. Iran. Chem. Soc..

[61] Mendes R.L., Fernandes H.L., Coelho J., Reis E.C., Cabral J.M., Novais J.M., Palavra A.F. (1995). Supercritical CO_2_ extraction of carotenoids and other lipids from *Chlorella vulgaris*. Food Chem..

[62] Mendes R.L., Coelho J.P., Fernandes H.L., Marrucho I.J., Cabral J.M.S., Novais J.M., Palavra A.F. (1995). Applications of supercritical CO_2_ extraction to microalgae and plants. J. Chem. Technol. Biotechnol..

[63] Obeid S., Beaufils N., Camy S., Takache H., Ismail A., Pontalier P.-Y. (2018). Supercritical carbon dioxide extraction and fractionation of lipids from freeze-dried microalgae *Nannochloropsis oculata* and *Chlorella vulgaris*. Algal Res..

[64] Ruen-ngam D., Shotipruk A., Pavasant P., Machmudah S., Goto M. (2012). Selective Extraction of Lutein from Alcohol Treated *Chlorella vulgaris* by Supercritical CO_2_. Chem. Eng. Technol..

[65] Wang H.-M., Pan J.-L., Chen C.-Y., Chiu C.-C., Yang M.-H., Chang H.-W., Chang J.-S. (2010). Identification of anti-lung cancer extract from Chlorella vulgaris C-C by antioxidant property using supercritical carbon dioxide extraction. Process Biochem..

[66] Georgiopoulou I., Tzima S., Louli V., Magoulas K. (2022). Supercritical CO2 Extraction of High-Added Value Compounds from *Chlorella vulgaris*: Experimental Design, Modelling and Optimization. Molecules.

[67] khorramdashti Mohammad S., Giri Mohammad S., Majidian N. (2021). Extraction lipids from chlorella vulgaris by supercritical CO_2_ for biodiesel production. S. Afr. J. Chem. Eng..

[68] Halim R., Gladman B., Danquah M.K., Webley P.A. (2011). Oil extraction from microalgae for biodiesel production. Bioresour. Technol..

[69] Ota M., Watanabe H., Kato Y., Watanabe M., Sato Y., Smith R.L., Inomata H. (2009). Carotenoid production from Chlorococcum littorale in photoautotrophic cultures with downstream supercritical fluid processing. J. Sep. Sci..

[70] Chen K.T., Cheng C.H., Wu Y.H., Lu W.C., Lin Y.H., Lee H.T. (2013). Continuous lipid extraction of microalgae using high-pressure carbon dioxide. Bioresour. Technol..

[71] Couto R.M., Simões P.C., Reis A., Da Silva T.L., Martins V.H., Sánchez-Vicente Y. (2010). Supercritical fluid extraction of lipids from the heterotrophic microalga *Crypthecodinium cohnii*. Eng. Life Sci..

[72] Jaime L., Mendiola J.A., Ibáñez E., Martin-Álvarez P.J., Cifuentes A., Reglero G., Señoráns F.J. (2007). β-Carotene Isomer Composition of Sub- and Supercritical Carbon Dioxide Extracts. Antioxidant Activity Measurement. J. Agric. Food Chem..

[73] Mendiola J.A., Santoyo S., Cifuentes A., Reglero G., IbÁÑEz E., SeÑOrÁNs F.J. (2008). Antimicrobial Activity of Sub- and Supercritical CO_2_ Extracts of the Green Alga *Dunaliella salina*. J. Food Prot..

[74] Macias-Sanchez M.D., Mantell C., Rodriguez M., Martinez de la Ossa E., Lubian L.M., Montero O. (2009). Comparison of supercritical fluid and ultrasound-assisted extraction of carotenoids and chlorophyll a from *Dunaliella salina*. Talanta.

[75] Macías-Sánchez M.D., Serrano C.M., Rodríguez M.R., Martínez de la Ossa E. (2009). Kinetics of the supercritical fluid extraction of carotenoids from microalgae with CO_2_ and ethanol as cosolvent. Chem. Eng. J..

[76] Macias-Sanchez M.D., Mantell Serrano C., Rodriguez M.R., Martinez de la Ossa E., Lubian L.M., Montero O. (2008). Extraction of carotenoids and chlorophyll from microalgae with supercritical carbon dioxide and ethanol as cosolvent. J. Sep. Sci..

[77] Pour Hosseini S.R., Tavakoli O., Sarrafzadeh M.H. (2017). Experimental optimization of SC-CO_2_ extraction of carotenoids from *Dunaliella salina*. J. Supercrit. Fluids.

[78] Molino A., Larocca V., Di Sanzo G., Martino M., Casella P., Marino T., Karatza D., Musmarra D. (2019). Extraction of Bioactive Compounds Using Supercritical Carbon Dioxide. Molecules.

[79] Tirado D.F., Calvo L. (2019). The Hansen theory to choose the best cosolvent for supercritical CO_2_ extraction of β-carotene from *Dunaliella salina*. J. Supercrit. Fluids.

[80] Yothipitak W., Goto M., Shotipruk A. (2008). Experiments and Statistical Analysis of Supercritical Carbon Dioxide Extraction. Chiang Mai J. Sci..

[81] Thana P., Machmudah S., Goto M., Sasaki M., Pavasant P., Shotipruk A. (2008). Response surface methodology to supercritical carbon dioxide extraction of astaxanthin from *Haematococcus pluvialis*. Bioresour. Technol..

[82] Bustamante A., Roberts P.J., Aravena R.I., Valle J.M.d. Supercritical extraction of astaxanthin from *H. pluvialis* using ethanol-modified CO_2_. Experiments and modeling. Proceedings of the 11th International Conference of Eng Food.

[83] Pan J.-L., Wang H.-M., Chen C.-Y., Chang J.-S. (2012). Extraction of astaxanthin from *Haematococcus pluvialis* by supercritical carbon dioxide fluid with ethanol modifier. Eng. Life Sci..

[84] Aravena R.I., del Valle J.M. Effect of microalgae preconditioning on supercritical CO_2_ extraction of astaxanthin from *Haematococcus pluvialis*. Proceedings of the 10th International Symposium of Supercritical Fluids.

[85] Kwan T.A., Kwan S.E., Peccia J., Zimmerman J.B. (2018). Selectively biorefining astaxanthin and triacylglycerol co-products from microalgae with supercritical carbon dioxide extraction. Bioresour. Technol..

[86] Nobre B., Marcelo F., Passos R., Beirão L., Palavra A., Gouveia L., Mendes R. (2006). Supercritical carbon dioxide extraction of astaxanthin and other carotenoids from the microalga *Haematococcus pluvialis*. Eur. Food Res. Technol..

[87] Machmudah S., Shotipruk A., Goto M., Sasaki M., Hirose T. (2006). Extraction of Astaxanthin from *Haematococcus pluvialis* Using Supercritical CO_2_ and Ethanol as Entrainer. Ind. Eng. Chem. Res..

[88] Krichnavaruk S., Shotipruk A., Goto M., Pavasant P. (2008). Supercritical carbon dioxide extraction of astaxanthin from *Haematococcus pluvialis* with vegetable oils as co-solvent. Bioresour. Technol..

[89] Wang L., Yang B., Yan B., Yao X. (2012). Supercritical fluid extraction of astaxanthin from *Haematococcus pluvialis* and its antioxidant potential in sunflower oil. Innov. Food Sci. Emerg. Technol..

[90] Reyes F.A., Mendiola J.A., Ibañez E., del Valle J.M. (2014). Astaxanthin extraction from *Haematococcus pluvialis* using CO_2_-expanded ethanol. J. Supercrit. Fluids.

[91] Sanzo G.D., Mehariya S., Martino M., Larocca V., Casella P., Chianese S., Musmarra D., Balducchi R., Molino A. (2018). Supercritical Carbon Dioxide Extraction of Astaxanthin, Lutein, and Fatty Acids from *Haematococcus pluvialis* Microalgae. Mar. Drugs.

[92] Molino A., Mehariya S., Iovine A., Larocca V., Di Sanzo G., Martino M., Casella P., Chianese S., Musmarra D. (2018). Extraction of Astaxanthin and Lutein from Microalga *Haematococcus pluvialis* in the Red Phase Using CO(_2_) Supercritical Fluid Extraction Technology with Ethanol as Co-Solvent. Mar. Drugs.

[93] Hernández D., Solana M., Riaño B., García-González M.C., Bertucco A. (2014). Biofuels from microalgae: Lipid extraction and methane production from the residual biomass in a biorefinery approach. Bioresour. Technol..

[94] Gilbert-López B., Mendiola J.A., Fontecha J., van den Broek L.A.M., Sijtsma L., Cifuentes A., Herrero M., Ibáñez E. (2015). Downstream processing of *Isochrysis galbana*: A step towards microalgal biorefinery. Green Chem..

[95] Fujii K. (2012). Process integration of supercritical carbon dioxide extraction and acid treatment for astaxanthin extraction from a vegetative microalga. Food Bioprod. Process..

[96] Macías-Sánchez M.D., Mantell C., Rodríguez M., Martínez de la Ossa E., Lubián L.M., Montero O. (2005). Supercritical fluid extraction of carotenoids and chlorophyll a from *Nannochloropsis gaditana*. J. Food Eng..

[97] Sánchez-Camargo A.d.P., Pleite N., Mendiola J.A., Cifuentes A., Herrero M., Gilbert-López B., Ibáñez E. (2018). Development of green extraction processes for *Nannochloropsis gaditana* biomass valorization. Electrophoresis.

[98] Molino A., Martino M., Larocca V., Di Sanzo G., Spagnoletta A., Marino T., Karatza D., Iovine A., Mehariya S., Musmarra D. (2019). Eicosapentaenoic Acid Extraction from *Nannochloropsis gaditana* using Carbon Dioxide at Supercritical Conditions. Mar. Drugs.

[99] Bjornsson W.J., MacDougall K.M., Melanson J.E., O’Leary S.J.B., McGinn P.J. (2011). Pilot-scale supercritical carbon dioxide extractions for the recovery of triacylglycerols from microalgae: A practical tool for algal biofuels research. J. Appl. Phycol..

[100] Tibbetts S.M., Bjornsson W.J., McGinn P.J. (2015). Biochemical composition and amino acid profiles of *Nannochloropsis granulata* algal biomass before and after supercritical fluid CO_2_ extraction at two processing temperatures. Anim. Feed Sci. Technol..

[101] Liau B.-C., Shen C.-T., Liang F.-P., Hong S.-E., Hsu S.-L., Jong T.-T., Chang C.-M.J. (2010). Supercritical fluids extraction and anti-solvent purification of carotenoids from microalgae and associated bioactivity. J. Supercrit. Fluids.

[102] Crampon C., Mouahid A., Toudji S.-A.A., Lépine O., Badens E. (2013). Influence of pretreatment on supercritical CO_2_ extraction from *Nannochloropsis oculata*. J. Supercrit. Fluids.

[103] Bong S.C., Loh S. (2013). A study of fatty acid composition and tocopherol content of lipid extracted from marine microalgae, *Nannochloropsis oculata* and *Tetraselmis suecica*, using solvent extraction and supercritical fluid extraction. Int. Food Res. J..

[104] Andrich G., Nesti U., Venturi F., Zinnai A., Fiorentini R. (2005). Supercritical fluid extraction of bioactive lipids from the microalga *Nannochloropsis* sp. Eur. J. Lipid Sci. Technol..

[105] Nobre B.P., Villalobos F., Barragan B.E., Oliveira A.C., Batista A.P., Marques P.A., Mendes R.L., Sovova H., Palavra A.F., Gouveia L. (2013). A biorefinery from *Nannochloropsis* sp. microalga—Extraction of oils and pigments. Production of biohydrogen from the leftover biomass. Bioresour. Technol..

[106] Leone G.P., Balducchi R., Mehariya S., Martino M., Larocca V., Di Sanzo G., Iovine A., Casella P., Marino T., Karatza D. (2019). Selective Extraction of ω-3 Fatty Acids from *Nannochloropsis* sp. using Supercritical CO_2_ Extraction. Molecules.

[107] Polak J.T., Balaban M., Peplow A., Phlips A.J. (1989). Supercritical Carbon Dioxide Extraction of Lipids from Algae. Supercritical Fluid Science and Technology.

[108] Cheng C.H., Du T.B., Pi H.C., Jang S.M., Lin Y.H., Lee H.T. (2011). Comparative study of lipid extraction from microalgae by organic solvent and supercritical CO_2_. Bioresour. Technol..

[109] Tommasi E., Cravotto G., Galletti P., Grillo G., Mazzotti M., Sacchetti G., Samorì C., Tabasso S., Tacchini M., Tagliavini E. (2017). Enhanced and Selective Lipid Extraction from the Microalga *P. tricornutum* by Dimethyl Carbonate and Supercritical CO_2_ using Deep Eutectic Solvents and Microwaves as Pretreatment. ACS Sustain. Chem. Eng..

[110] Chatterjee D., Bhattacharjee P. (2014). Supercritical carbon dioxide extraction of antioxidant rich fraction from *Phormidium valderianum*: Optimization of experimental process parameters. Algal Res..

[111] Macías-Sánchez M.D., Fernandez-Sevilla J.M., Fernández F.A., García M.C., Grima E. (2010). Supercritical fluid extraction of carotenoids from *Scenedesmus almeriensis*. Food Chem..

[112] Mehariya S., Iovine A., Di Sanzo G., Larocca V., Martino M., Leone G.P., Casella P., Karatza D., Marino T., Musmarra D. (2019). Supercritical Fluid Extraction of Lutein from *Scenedesmus almeriensis*. Molecules.

[113] Soh L., Zimmerman J. (2011). Biodiesel production: The potential of algal lipids extracted with supercritical carbon dioxide. Green Chem..

[114] Gilbert-López B., Mendiola J.A., van den Broek L.A.M., Houweling-Tan B., Sijtsma L., Cifuentes A., Herrero M., Ibáñez E. (2017). Green compressed fluid technologies for downstream processing of *Scenedesmus obliquus* in a biorefinery approach. Algal Res..

[115] Choi K.J., Nakhost Z., Krukonis V.J., Karel M. (1987). Supercritical fluid extraction and characterization of lipids from algae *Scenedesmus obliquus*. Food Biotechnol..

[116] Guedes A.C., Gião M.S., Matias A.A., Nunes A.V.M., Pintado M.E., Duarte C.M.M., Malcata F.X. (2013). Supercritical fluid extraction of carotenoids and chlorophylls a, b and c, from a wild strain of *Scenedesmus obliquus* for use in food processing. J. Food Eng..

[117] Klejdus B., Lojkova L., Plaza M., Snoblova M., Sterbova D. (2010). Hyphenated technique for the extraction and determination of isoflavones in algae: Ultrasound-assisted supercritical fluid extraction followed by fast chromatography with tandem mass spectrometry. J. Chromatogr. A.

[118] Yen H.-W., Chiang W.-C., Sun C.-H. (2012). Supercritical fluid extraction of lutein from Scenedesmus cultured in an autotrophical photobioreactor. J. Taiwan Inst. Chem. Eng..

[119] Abrahamsson V., Rodriguez-Meizoso I., Turner C. (2012). Determination of carotenoids in microalgae using supercritical fluid extraction and chromatography. J. Chromatogr. A.

[120] Taher H., Al-Zuhair S., Al-Marzouqi A.H., Haik Y., Farid M., Tariq S. (2014). Supercritical carbon dioxide extraction of microalgae lipid: Process optimization and laboratory scale-up. J. Supercrit. Fluids.

[121] Shomal R., Hisham H., Mlhem A., Hassan R., Al-Zuhair S. (2019). Simultaneous extraction–reaction process for biodiesel production from microalgae. Energy Rep..

[122] Montero O., Macías-Sánchez M.D., Lama C.M., Lubián L.M., Mantell C., Rodríguez M., de la Ossa E.M. (2005). Supercritical CO_2_ extraction of beta-carotene from a marine strain of the cyanobacterium Synechococcus species. J. Agric. Food Chem..

[123] Macías-Sánchez M.D., Mantell C., Rodríguez M., Martínez de la Ossa E., Lubián L.M., Montero O. (2007). Supercritical fluid extraction of carotenoids and chlorophyll a from *Synechococcus* sp. J. Supercrit. Fluids.

[124] Grierson S., Strezov V., Bray S., Mummacari R., Danh L.T., Foster N. (2012). Assessment of Bio-oil Extraction from Tetraselmis chui Microalgae Comparing Supercritical CO_2_, Solvent Extraction, and Thermal Processing. Energy Fuels.

[125] Li Y., Ghasemi Naghdi F., Garg S., Adarme-Vega T.C., Thurecht K.J., Ghafor W.A., Tannock S., Schenk P.M. (2014). A comparative study: The impact of different lipid extraction methods on current microalgal lipid research. Microb. Cell Factories.

[126] Safafar H., van Wagenen J., Moller P., Jacobsen C. (2015). Carotenoids, Phenolic Compounds and Tocopherols Contribute to the Antioxidative Properties of Some Microalgae Species Grown on Industrial Wastewater. Mar. Drugs.

[127] Hosikian A., Lim S., Halim R., Danquah M.K. (2010). Chlorophyll Extraction from Microalgae: A Review on the Process Engineering Aspects. Int. J. Chem. Eng..

[128] Fu W., Nelson D.R., Yi Z., Xu M., Khraiwesh B., Jijakli K., Chaiboonchoe A., Alzahmi A., Al-Khairy D., Brynjolfsson S., Attaur R. (2017). Chapter 6 - Bioactive Compounds from Microalgae: Current Development and Prospects. Studies in Natural Products Chemistry.

[129] Balasubramaniam V., Gunasegavan R.D., Mustar S., Lee J.C., Mohd Noh M.F. (2021). Isolation of Industrial Important Bioactive Compounds from Microalgae. Molecules.

[130] Novoveská L., Ross M.E., Stanley M.S., Pradelles R., Wasiolek V., Sassi J.F. (2019). Microalgal Carotenoids: A Review of Production, Current Markets, Regulations, and Future Direction. Mar. Drugs.

[131] Guedes A.C., Amaro H.M., Malcata F.X. (2011). Microalgae as Sources of Carotenoids. Mar. Drugs.

[132] Smaoui S., Barkallah M., Ben Hlima H., Fendri I., Mousavi Khaneghah A., Michaud P., Abdelkafi S. (2021). Microalgae Xanthophylls: From Biosynthesis Pathway and Production Techniques to Encapsulation Development. Foods.

[133] Henríquez V., Escobar C., Galarza J., Gimpel J. (2016). Carotenoids in microalgae. Carotenoids Nat..

[134] da Silva J.C., Lombardi A.T., Jacob-Lopes E., Queiroz M.I., Zepka L.Q. (2020). Chlorophylls in Microalgae: Occurrence, Distribution, and Biosynthesis. Pigments from Microalgae Handbook.

[135] da Silva Ferreira V., Sant’Anna C. (2016). Impact of culture conditions on the chlorophyll content of microalgae for biotechnological applications. World J. Microbiol. Biotechnol..

[136] Mimouni V., Couzinet-Mossion A., Ulmann L., Wielgosz-Collin G., Levine I.A., Fleurence J. (2018). Chapter 5—Lipids from Microalgae. Microalgae in Health and Disease Prevention.

[137] de Carvalho J.C., Magalhaes A.I., de Melo Pereira G.V., Medeiros A.B.P., Sydney E.B., Rodrigues C., Aulestia D.T.M., de Souza Vandenberghe L.P., Soccol V.T., Soccol C.R. (2020). Microalgal biomass pretreatment for integrated processing into biofuels, food, and feed. Bioresour. Technol..

[138] Lee S.Y., Cho J.M., Chang Y.K., Oh Y.K. (2017). Cell disruption and lipid extraction for microalgal biorefineries: A review. Bioresour. Technol..

[139] Bernaerts T.M.M., Gheysen L., Foubert I., Hendrickx M.E., Van Loey A.M. (2019). The potential of microalgae and their biopolymers as structuring ingredients in food: A review. Biotechnol. Adv..

[140] Lee A.K., Lewis D.M., Ashman P.J. (2012). Disruption of microalgal cells for the extraction of lipids for biofuels: Processes and specific energy requirements. Biomass Bioenergy.

[141] Postma P.R., Suarez-Garcia E., Safi C., Yonathan K., Olivieri G., Barbosa M.J., Wijffels R.H., Eppink M.H.M. (2017). Energy efficient bead milling of microalgae: Effect of bead size on disintegration and release of proteins and carbohydrates. Bioresour. Technol..

[142] Suarez Garcia E., Lo C., Eppink M.H.M., Wijffels R.H., van den Berg C. (2019). Understanding mild cell disintegration of microalgae in bead mills for the release of biomolecules. Chem. Eng. Sci..

[143] Greenly J.M., Tester J.W. (2015). Ultrasonic cavitation for disruption of microalgae. Bioresour. Technol..

[144] Pan J., Muppaneni T., Sun Y., Reddy H.K., Fu J., Lu X., Deng S. (2016). Microwave-assisted extraction of lipids from microalgae using an ionic liquid solvent [BMIM][HSO4]. Fuel.

[145] Passos F., Uggetti E., Carrère H., Ferrer I. (2015). Algal Biomass. Pretreatment of Biomass.

[146] D’Hondt E., Martín-Juárez J., Bolado S., Kasperoviciene J., Koreiviene J., Sulcius S., Elst K., Bastiaens L., Gonzalez-Fernandez C., Muñoz R. (2017). 6—Cell disruption technologies. Microalgae-Based Biofuels and Bioproducts.

[147] Yoo G., Park M.S., Yang J.-W. (2015). Chemical Pretreatment of Algal Biomass. Pretreatment of Biomass.

[148] Dular M., Griessler-Bulc T., Gutierrez-Aguirre I., Heath E., Kosjek T., Krivograd Klemencic A., Oder M., Petkovsek M., Racki N., Ravnikar M. (2016). Use of hydrodynamic cavitation in (waste) water treatment. Ultrason. Sonochem..

[149] Sun X., Liu J., Ji L., Wang G., Zhao S., Yoon J.Y., Chen S. (2020). A review on hydrodynamic cavitation disinfection: The current state of knowledge. Sci. Total Environ..

[150] Lam G.P.t., Postma P.R., Fernandes D.A., Timmermans R.A.H., Vermuë M.H., Barbosa M.J., Eppink M.H.M., Wijffels R.H., Olivieri G. (2017). Pulsed Electric Field for protein release of the microalgae *Chlorella vulgaris* and *Neochloris oleoabundans*. Algal Res..

[151] Zbinden M.D., Sturm B.S., Nord R.D., Carey W.J., Moore D., Shinogle H., Stagg-Williams S.M. (2013). Pulsed electric field (PEF) as an intensification pretreatment for greener solvent lipid extraction from microalgae. Biotechnol. Bioeng..

[152] Mishra V., Dubey A., Prajapti S.K. (2017). Algal Biomass Pretreatment for Improved Biofuel Production. Algal Biofuels.

[153] Lorente E., Haponska M., Clavero E., Torras C., Salvado J. (2017). Microalgae fractionation using steam explosion, dynamic and tangential cross-flow membrane filtration. Bioresour. Technol..

[154] Aarthy A., Kumari S., Turkar P., Subramanian S. (2018). An insight on algal cell disruption for biodiesel production. Asian J. Pharm. Clin. Res..

[155] Velazquez-Lucio J., Rodríguez-Jasso R.M., Colla L.M., Sáenz-Galindo A., Cervantes-Cisneros D.E., Aguilar C.N., Fernandes B.D., Ruiz H.A. (2018). Microalgal biomass pretreatment for bioethanol production: A review. Biofuel Res. J..

[156] Khoo K.S., Lee S.Y., Ooi C.W., Fu X., Miao X., Ling T.C., Show P.L. (2019). Recent advances in biorefinery of astaxanthin from *Haematococcus pluvialis*. Bioresour. Technol..

[157] Scholz M.J., Weiss T.L., Jinkerson R.E., Jing J., Roth R., Goodenough U., Posewitz M.C., Gerken H.G. (2014). Ultrastructure and Composition of the Nannochloropsis gaditana Cell Wall. Eukaryot. Cell.

[158] Mularczyk M., Michalak I., Marycz K. (2020). Astaxanthin and other Nutrients from *Haematococcus pluvialis*—Multifunctional Applications. Mar. Drugs.

[159] Burczyk J., Szkawran H., Zontek I., Czygan F.-C. (1981). Carotenoids in the Outer Cell-Wall Layer of Scenedesmus (*Chlorophyceae*). Planta.

[160] Kopcak U., Mohamed R.S. (2005). Caffeine solubility in supercritical carbon dioxide/co-solvent mixtures. J. Supercrit. Fluids.

[161] Sovová H. (2005). Mathematical model for supercritical fluid extraction of natural products and extraction curve evaluation. J. Supercrit. Fluids.

[162] Oliveira E.L.G., Silvestre A.J.D., Silva C.M. (2011). Review of kinetic models for supercritical fluid extraction. Chem. Eng. Res. Des..

[163] Sovová H. (1994). Rate of the vegetable oil extraction with supercritical CO_2_—I. Modelling of extraction curves. Chem. Eng. Sci..

[164] Huang Z., Shi X.-h., Jiang W.-j. (2012). Theoretical models for supercritical fluid extraction. J. Chromatogr. A.

[165] Folch J., Lees M., Sloane Stanley G.H. (1957). A simple method for the isolation and purification of total lipids from animal tissues. J Biol. Chem..

[166] Bligh E.G., Dyer W.J. (1959). A rapid method of total lipid extraction and purification. Can. J. Biochem. Physiol..

[167] Iverson S.J., Lang S.L.C., Cooper M.H. (2001). Comparison of the bligh and dyer and folch methods for total lipid determination in a broad range of marine tissue. Lipids.

[168] Araujo G.S., Matos L.J.B.L., Fernandes J.O., Cartaxo S.J.M., Gonçalves L.R.B., Fernandes F.A.N., Farias W.R.L. (2013). Extraction of lipids from microalgae by ultrasound application: Prospection of the optimal extraction method. Ultrason. Sonochemistry.

[169] Kaufmann B., Christen P. (2002). Recent extraction techniques for natural products: Microwave-assisted extraction and pressurised solvent extraction. Phytochem. Anal..

[170] Veggi P.C., Martinez J., Meireles M.A.A., Chemat F., Cravotto G. (2013). Fundamentals of Microwave Extraction. Microwave-assisted Extraction for Bioactive Compounds: Theory and Practice.

[171] Mandal V., Mohan Y., Hemalatha S. (2006). Microwave Assisted Extraction—An Innovative and Promising Extraction Tool for Medicinal Plant Research. Pharmacogn. Rev..

